# Conformational Flexibility of Transmembrane Helices: How it Works and Where it Matters

**DOI:** 10.1021/acs.chemrev.5c00581

**Published:** 2026-02-25

**Authors:** Dieter Langosch

**Affiliations:** Technical University of Munich, School of Life Sciences, Alte Akademie 8, 85354 Freising, Germany

## Abstract

An increasing number of multipass and oligomeric membrane proteins is found to exist in different structural substates that represent different stages of their functional cycles. Many of their constituent transmembrane helices locally deviate from canonical α–helical structure, suggesting that their conformational flexibility is required for function and/or connected to structural conversions between functional states. Biological functions of many single-pass proteins also often depend on the substantial conformational flexibility of their transmembrane helices. Current research focuses on the types and sequence dependence of helix flexibility, its diverse functional roles, as well as its interplay with the lipid environment within a membrane. This Perspective will illustrate these issues using a number of exemplary cases, including bacteriorhodopsin, ion channels, fusogenic proteins, and intramembrane protease substrates. In addition, we will discuss some methodological aspects, including advanced hydrogen–deuterium exchange analysis that can be useful in investigating the conformational flexibility of TM-helices.

## Graphical abstract

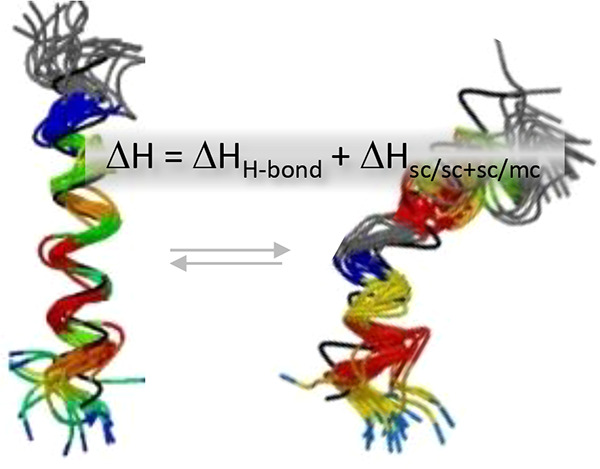

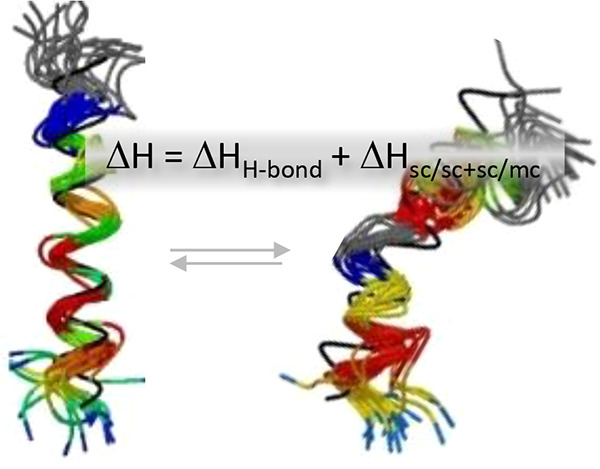

## 1 Introduction

The first low-resolution 3D-structure of the multipass membrane protein bacteriorhodopsin (bR) solved by Henderson and Unwin in 1975 made it clear that this protein traverses the lipid bilayer by way of seven helices.^1^ Although β–barrels were soon to be known as alternate way by which a polypeptide chain can cross a bilayer,^2^ the concept of straight α–helices spanning the membrane shaped our understanding of integral membrane protein structure for decades. Soon, tools to predict transmembrane (TM) helices and TM-topology from primary structure routinely produced topology models for a wide diversity of integral membrane proteins.^3^ This enabled Brandl and Deber as early as 1986 to show that Pro residues are much more abundant within predicted transmembrane domains (TMDs) of nearly all transport proteins then examined than within the TMDs of nontransport proteins.^4^ At the time, this was taken as evidence that membrane-buried Pro residues support the transporter function. Also, Pro was already known as a potent helix breaker^5^ and among the rarest and thus presumably most helix-destabilizing amino acids in globular proteins.^6^ Its relative enrichment in TMDs of transport proteins thus suggested that local helix distortion plays a role in the conformational changes during opening and closing of transport channels.^4^ Decades later, progress in high-resolution structure determination unveiled numerous membrane proteins to exist in alternative conformations assumed at different stages of their functional cycles. The corresponding structural changes are characterized by a range of magnitudes and time-scales. They follow a hierarchy ranging from low-amplitude fluctuations of individual atoms around their mean positions in a TM-helix to altered geometries of helix–helix packing. Many natural mutations in membrane proteins can cause hereditary diseases. Underscoring the concept of functionally relevant conformational flexibility, these mutations mostly occur within TM-helices and coil regions where they usually increase protein dynamics at different levels of the hierarchy.^7^ In terms of its physical underpinnings, the conformational dynamics of membrane proteins is similar to that of their soluble counterparts.^7–9^ However, membrane proteins can be subject to various constraints exerted by the lipid bilayer via a number of different mechanisms, of which most are still incompletely understood.

By covering about three decades of research, this review will take a close look at different aspects of these issues. In the “how it works” part of the text, we will look into the detection of non-α-helical elements by geometric analysis within TM-helices of 3D-structures, as well as into interhelical packing densities. Evidence linking these structural aspects to conformational dynamics will be discussed. Further, we will scrutinize a wide range of model studies where early attempts of protein design contributed to the physicochemical basis of how primary structure determines conformational flexibility. Taken a scientific impressionist’s stance, I will then try to illustrate the theoretical issues by examining their biological consequences using a number of paradigmatic model cases. This “where it matters” part includes light-driven proton transport by bR, ion flux through channel proteins, lipid membrane fusion, and intramembrane proteolysis. Among the methods most commonly used to investigate helix dynamics (reviewed in ref 10), the use of hydrogen/deuterium exchange will be alluded to in some detail. The terms conformational “flexibility” and “dynamics” will be used synonymously.

## 2 The Conformational Dynamics of Multipass Membrane Proteins

To start our journey, evidence for structural aberrations in known membrane protein 3D structures will be presented. These findings will be connected to the overrepresentation of certain amino acid types within these noncanonical regions and to mechanisms of how these regions contribute to structural dynamics. Paradigmatic examples, including a 7TM-helix receptor and different ion channel proteins, will illustrate how such aberrations can contribute to the structural transitions that are commonly associated with the cycling between functional states.

### 2.1 3D-Structures Reveal Deviations from Canonical α-Helicity in TMDs

The canonical α-helix is defined by (i,i-4) main-chain H-bonding that extends from an amide nitrogen at residue (i) to the carbonyl oxygen at residue (i-4). This results in 3.6 residues per helical turn, a spoke angle of 100°, and a rise-per-residue of 1.54 Å, as intuitively predicted by Corey and Pauling based on geometrical considerations.^11^ The geometry of a helix is also described by the mean φ and ψ main-chain torsion angles of the Ramachandran plot. Naturally occurring α-helices exhibit average values of φ = −62° and ψ = −41°, that deviate slightly from the idealized helix and result from maximizing main-chain H-bonding.^12^ Helix termini show “fraying” of the main chain, an increase in conformational flexibility that is characterized by a larger variance of torsion angles compared to the core of a helix (Figure 1 A). Fraying results from nonbonded backbone amides and carbonyl oxygens within the final helical turns at N-termini and C-termini, respectively.^13^ TM-helices of multipass proteins and helices of globular proteins have been compared with respect to the amino acid compositions at their termini. The comparison revealed that both types of helices share an over-representation of polar amino acids and Pro near their N-termini as well as Gly and Pro near C-termini. Briefly, these overrepresented amino acids appear to terminate a helix via complex side-chain/main-chain interactions resulting in a variety of capping motifs.^9,14,15^ TM-helices are distinguished by an additional accumulation of Gly and His near N-termini as well as of Gln, Lys, and Cys near C-termini pointing to a modulating influence of the lipid membrane.^16^

**Figure 1:**
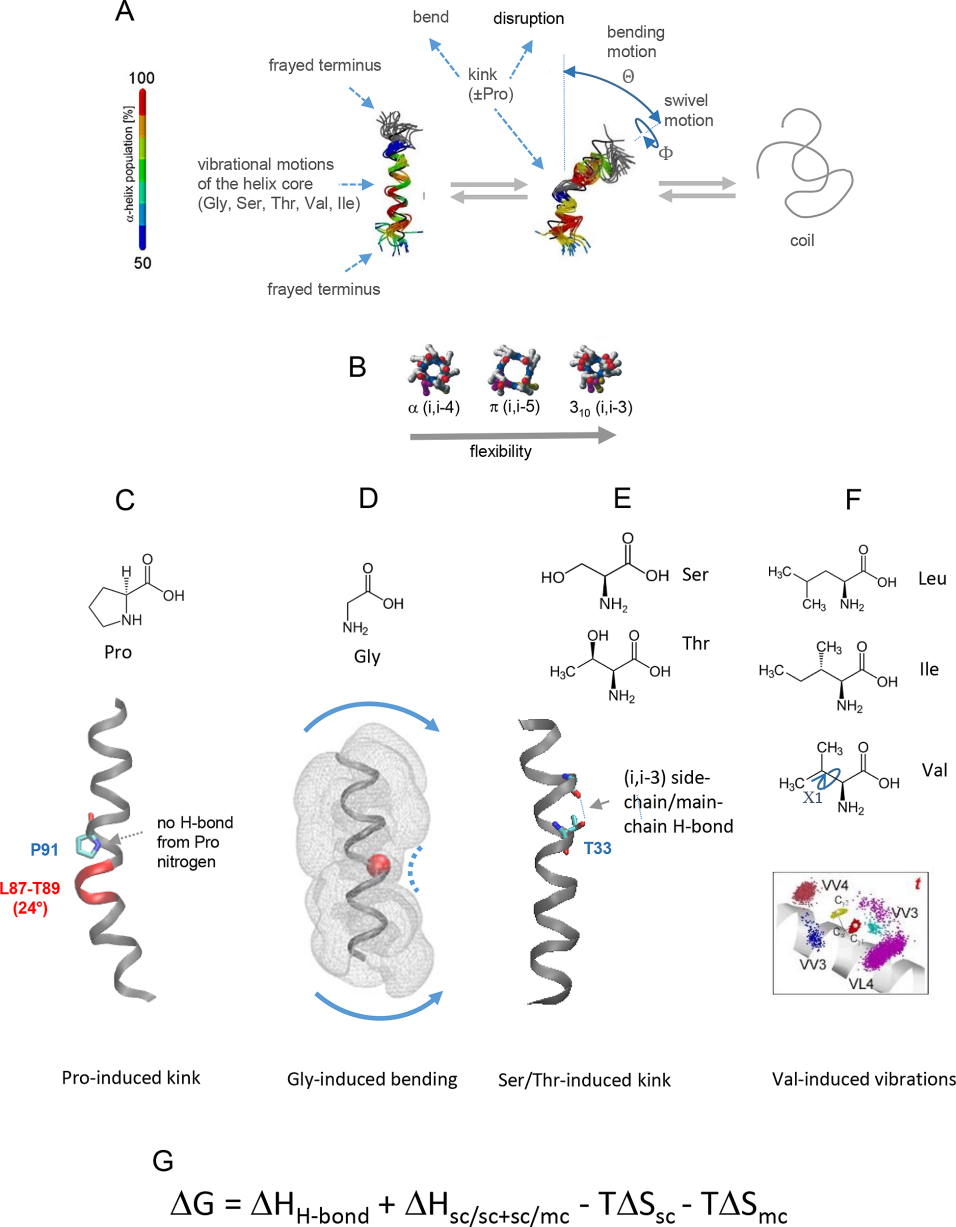
Deviations from α-helicity and TM-helix flexibility. (A) Common deviations from canonical α-helicity. Extended coil formation does usually not occur in hydrophobic environments and does not necessarily involve a kinked intermediate. The color code indicates the average population of α-helical H-bonds. (B) Geometry and stability of α-, π-, and 3_10_-helices (viewed from their N-termini). (C–F) Exemplary illustrations of how crucial amino acid types can induce local distortions. (C) Pro-induced kinking results from lack of amide H-bonding from the cyclic P91 side chain at (i) to (i-3,4) main-chain carbonyls, as exemplified by bR helix C (pdb 1C3W^20^). The identities of the kink residues (in red) and the kink angle were determined by MC-HELAN.^21^ (D) Gly-induced bending (indicated by curved arrows) of a model helix, as indicated by MD simulation,^22^ results from a cavity at Gly (indicated by the curved dotted line). The space that is explored by the helix surface in the course of its MD trajectory is indicated by dots. (E) Thr-induced kinking is thought to result from (i,i-3,4) H-bonding of the Thr side chain to a main-chain carbonyl oxygen, as exemplified by potassium channel helix 1 (pdb 1BL8). (F) Val can permit local fluctuations of a helix as its small side chain is essentially restricted to the *trans* rotamer in a helix (Χ1 = 180°). Therefore, C_γ_ methyl groups of a Val at position (i) can only form weak intrahelical interactions with the side chains of other residues at positions (i ± 3) and (i ± 4).^23^ See Figure 6H for the stronger interactions experienced by a Leu residue. (G) Thermodynamics of TM-helix formation. The Gibbs free energy Δ*G* of a helix consists of enthalphic (Δ*H*) and entropic (Δ*S*) contributions. Δ*H* includes strong amide H-bonding in the low dielectric plus supporting side-chain/side-chain (sc/sc) and side-chain/main chain (sc/mc) packing interactions. Δ*S* includes the loss of side-chain as well as of main-chain disorder in the course of helix formation (see NOTES below). For the sake of simplicity, the potential contributions of lipids to helix stability are excluded here. (A,F) Reproduced with permission from ref 23. Copyright 2010 Elsevier BV. (D) Reproduced with permission from ref 24. Copyright 2018 Americal Chemical Society. NOTES ON THE THEORY OF HELIX FOLDING: Despite decades of research, mainly done on soluble helices, the quantitative understanding of how primary structure (de)­stabilizes a helix still suffers from uncertainty.^9^ In an unstructured peptide, most side-chains can adopt more than one rotameric state, these states are distinguished by the Χ1 angles of rotation around the Cα–Cβ bond (*trans* = *t* equals an Χ1 angle of 180°; *gauche* ^+^ = *g* ^+^, Χ1 = +60°; *gauche* ^–^ = *g* ^–^, Χ1 = −60°). Formation of a helix restricts the number of allowed rotameric states for the β-branched residues Val and Ile essentially to the *t* rotamer. This restriction of rotamers results from the presence of two y-carbons that would sterically clash with backbone carbonyl oxygens upon adopting the other rotamers.^25,26^ In the case of γ-branched Leu, two rotameric states (*t* and *g* ^–^) are populated in a helix. It was thus argued that the entropy loss upon helix formation is smaller with Leu than with Val, Ile, or Thr. Statistics seemed to support the importance of side-chain entropy since the extent of its loss correlated with the helix-destabilizing tendencies of eight amino acids in the helix–coil equilibrium.^25^ However, the correlation was much weaker when all 20 amino acids were included in the analysis.^27^ Moreover, the entropy loss is similar for Ala (Δ*TS* = 0 kcal/mol) and Leu (0.04 kcal/mol)^25^ and therefore does not explain the stronger empirical helix stabilization by Ala vs Leu in aqueous solution.^28–33^ Indeed, it was suggested later that only 20% of the entropy loss associated with folding of a model protein is accounted for by side-chain entropy.^34^ In an alternative view, primary structure affects helix stability by intrahelical packing interactions between side chains as well as between side chains and the main chain. This view is supported by the correlation between the stability of a host helix within T4 lysozyme with the buried hydrophobic area of amino acids which increases in the order of Val < Ile < Leu.^27,35^ This is in line with computed van der Waals’ interactions between side chains located at (i, i ± 3, and i ± 4) positions along LV-peptides. Due to the larger size and greater conformational freedom of the Leu side chain, interactions involving Leu exceed those that involve Val.^23^ Accordingly, the reduced enthalpy of Val–Val or Val–Leu side-chain/side-chain interactions relative to Leu–Leu interactions may explain how increasing Val–Leu ratios in LV-peptide helices cause stronger structural fluctuations (see Figure 7A–E).^23,36^ Support for the impact of intrahelical packing interactions on helix stability also comes from kinetic studies. On the one hand, the assumption that helix formation is hindered by unfavorable rotameric states^25,26,37,38^ predicts that the kinetics of helix folding from a coil would be restrained by β-branched residues having to find their preferred rotameric state prior to helix formation; this would lower the folding rate *k*_cl_. On the other hand, stronger intrahelical side-chain interactions are expected to reduce the *unfolding* rate *k*_op_. Using Ala-based host peptides and a fluorescence transfer approach, Kiefhaber and co-workers have indeed shown that it is *k*_op_ that decreases from frayed helix termini toward helix cores while *k*_on_ is rather constant.^39^ The same method also confirmed that Leu (and Ile) affect local folding kinetics, while Val does not. At the level of rate constants, Val increases the rate of unfolding, but not the rate of folding, relative to Leu, thus explaining the helix-destabilizing effect of Val in model peptides by its preferential effect on helix unfolding.^40^ This is in keeping with the reduced side-chain interactions of Val noted above. In other words, it appears that the relationship between primary structure of a helix and its vibrational motions mainly rests on side chain interactions.^41,42^ Since a fluctuating helix backbone can exist in many configurations, entropic forces are likely to make a minor contribution to its flexibility.

Marked conformational flexibility within TM-helices was initially indicated by anecdotal evidence from the earliest membrane protein structures. These structures revealed deviations from the geometry of the canonical α-helix, such as “kinks” where its long helix axis changes direction (Figure 1A).^17,18^ Another conspicuous feature of many TM-helices was the occasional occurrence of local 3_10_-helices and π-helices (Figure 1B).^19^ As the (i,i-3) amide H-bonding pattern of short 3_10_-helices reduces their diameter, they are also designated “tight turns”. The opposite is true for π-helices, where (i,i-5) H-bonding results in “wide turns”. As these local deviations are usually associated with the absence, breakage, or rearrangement of intrahelical H-bonds, they are conspicuous of local conformational flexibility.

Systematic analyses of the growing set of membrane protein structures confirmed these early observations and revealed an abundance of structural irregularities within TM-helices (algorithms used are described in refs 43–47 ). Depending on the sample set and evaluation criteria, one-half to two-thirds of TM-helices exhibit one or several kinks.^21,48–51^ A major fraction of kinks is classified as “bend” and a minor one as “disruption”. Within a bend, the helical character as such is maintained, although the α-helix may be transformed into a 3_10_-helix or an π-helix for one or two helical turns. A disruption is associated with loss of helicity at the site of the kink. A disruption may result from only few broken H-bonds or correspond to a more extended local coil region.^52^ While the kink angles of most bends vary from 10° to 20°, disruptions result in a greater variability of angles which tend to exceed the angles of bends.^21^ It should be noted that some local 3_10_-helices or π-helices do not cause bending of an α–helical backbone.

About one-third of kinks are found at or near a Pro residue (Table 1). Indeed, relative to all other positions in a TM-helix, Pro is the most overrepresented amino acid type within a helical turn C-terminal of a bend and at or near a disruption. Pro is therefore the residue type most closely linked to kink formation. Pro can destabilize a helix since the imide within its cyclic side-chain cannot H-bond to an upstream carbonyl oxygen of the polypeptide chain. Further, a steric clash between the Pro ring and the backbone carbonyl group at position i-1 is produced (Figure 1C). It is thus not surprising that the kink angles related to Pro tend to be larger than those in the absence of Pro.^21,46,49,51^ The N-terminal segment of a Pro-kinked helix tends to bend in a direction away from the side of the missing H-bond.^53^ To make things even more complicated, more than one-third of the TM helices that contain a Pro remain rather straight. In these cases, the loss of an H-bond near Pro causes either a wide turn or a tight turn, depending on the type of compensatory H-bonding.^48,50^ Although Pro is the amino acid that is most conspicuous to introduce a helix kink, it should be remembered that more than two-thirds of kinks are not associated with Pro. This prompts the question of how these non-Pro kinks originate. An evolutionary hypothesis has been proposed where a mutation to Pro may initially induce a kink. During evolution, the resulting packing defects would be repaired by further mutation to maintain a functionally relevant kink, thus resulting in non-Pro kinks.^54^ This hypothesis was challenged, however, by sequence alignments showing that only a small fraction of non-Pro kinks could be tentatively traced back to an extant Pro.^50^ Moreover, analyzing homologous membrane proteins from a much larger database revealed that loss of a Pro often also results in the loss of a kink or reduction of its angle. To be sure, kink angle conservation is usually associated with Pro conservation.^51^ Another explanation of how non-Pro kinks form may relate to the overrepresention of amino acids with polar and ionizable side-chains at kinks,^46^ most notably at disruptions^21,49,52^ (Table 1). The side chains of these residues may stabilize a locally disrupted helix by donating an intrahelical hydrogen bond to an unbonded carbonyl oxygen.

**Table 1:** Deviations from Canonical α-Helical Structure Detected in Integral Membrane Proteins

deviation	favorable amino acids	ref
frayed termini	Pro, Gly, His, Cys, polar amino acids	^16^
3_10_-helix^a^	Pro, Ser, Ala	^48^,^50^,^55^
π-helix^a^	Gly, Ile, Val, Thr, Leu^c^	^48^,^50^,^55^
kink^b^	Pro, Gly, Ser	^46^,^48^,^50^,^51^
bend	Pro, Asp, Lys	^21^,^49^,^56^
disruption/coil	Pro, Gly, His, Glu, Asn, Asp, Lys, Arg	^21^,^49^,^52^
curvature	not determined	^46^

a Riek et al.^48^ and Hall et al.^50^ simply indicate tight and wide turns at Pro residues not causing kinks, these are listed under 3_10_- and π-helix, respectively.

b Riek et al.,^48^ Hall et al.,^50^ Law et al.,^51^ and Kneissl et al.^46^ have not distinguished bend and disruption. The overrepresented amino acids taken from their statistics are therefore summarily given for the kink category that also contains bends and disruptions.

c The noted propensities reflect the annotation of helical states by DSSP and are somewhat different when STRIDE is used to evaluate the pdb structures.^55^

Findings regarding the potential involvement of Gly in helix kinking are controversial. TM-helices are usually not kinked at Gly.^57^ At kinks, Gly was found to be slightly enriched by some authors^46,49,52^ or enriched when being part of extended motifs such as GxxGxxxG or GxxxGxxG.^50^ Others did not detect an overabundance of Gly at kinks.^21,44^ These differences are likely to result from using different data sets and geometrical measures of kink formation.

Most Ser, Thr, and to a lesser extent also Cys, cause bending of soluble and membrane-spanning helices by 3° to 4°.^58,59^ Bending results if the side-chain heteroatom forms an intrahelical (i,i-3) or (i,i-4) H-bond with a main chain carbonyl oxygen and thus destabilizes a local amide H-bond (Figure 1 E).^60^ Helix bending then is thought to result from electrostatic repulsion between the heteroatom at (i) and the (i-3) carbonyl oxygen. In other words, repulsion is minimized by modest helix bending as well as by local opening of the helix turn preceding Ser, Thr (or Cys).^59^ In addition, the side-chain/main-chain backbonding of Ser and Thr (or Cys) can either increase or decrease a Pro-induced bend, depending on the local sequence.^61,62^ We note that TM-helix stabilization by Thr backbonding is also known.^63,64^ Finally, about one-fifth of TM-helices are significantly curved.^46^

Thus, certain amino acid types are preferred at deviations from ideal α-helicity. At the same time, these amino acids generally show a reduced relative abundance in TM-helices. For example, Pro is generally disfavored in TM-helices, relative to soluble domains, especially near the bilayer core.^65–67^ By comparison, Gly is distributed rather evenly over the entire length of a TM-helix.^65–69^ Interestingly, however, Pro and Gly are much more prevalent in TM-helices than in soluble helices.^70^ To explain this difference, one may ask: Why are Pro and Gly apparently better tolerated in apolar environments than in polar environments? Apolar environments, such as a lipid bilayer, are known to stabilize TM-helices since amide H-bonds formed by the helix backbone are stronger in the absence of water that can serve as alternative H-bond partner for a protein in the coil state.^71,72^ As a result, Pro and Gly can locally destabilize a membrane-embedded TM-helix. In the absence of water, they do not cause complete unfolding, however. Further, depending on their lipid composition, lipid bilayers exhibit a steep polarity gradient.^73^ For example, in the core of a phosphatidylcholine bilayer the permittivity ε equals ∼4 while it approaches ∼12 at the interface where acyl-chains are linked to the glycerol backbone.^74^ Consequently, a TM-helix is most stable at the bilayer core, where exposing potential H-bond partners is met with the greatest energetic penalty. This effect provides one explanation for the generally reduced abundance of Pro toward the bilayer core.^65–68^

We note that the concept of H-bond stabilization by apolar media has recently been questioned by proposing that the free energy of main-chain H-bond formation in a bilayer is independent of the depth of H-bond insertion and comparable to that in water-soluble proteins.^75^ Unfortunately, the hydrogen/deuterium fractionation factors used to determine H-bond strength in that study suffer from major inherent issues^64^ which questions the reported independence of H-bond strength^75^ on the polarity of the environment (see section 5 for details).

### 2.2 Irregularities in TM-Helix Structure Indicate Conformational Flexibility

How do these aberrations from idealized helix geometry contribute to a membrane protein’s conformational flexibility? Quite a number of membrane proteins are now known to adopt different structural substates that have mostly been obtained in the presence of specific agonists or antagonists or by introducing point mutations stabilizing certain conformations. Akin to the theory of allostery, as originally developed for soluble proteins,^76,77^ substates are commonly believed to represent distinct functional states, although apparent structural variability may also result from experimental conditions.^78^ Distinct substates often exhibit different geometries of their TM-helices. For example, the extent of TM-helix kinking differs between structures corresponding to distinct stages of Ca^2+^ transport by sarcoplasmic Ca^2+^-ATPase. To avoid the energetic penalty of H-bond breakage, kinking within the TM-helices of this transporter is compensated by interconversions between (i,i-3) and (i,i-4) H-bonding.^56^ As there is no disallowed region between α–helical and 3_10_-helical conformations in the Ramachandran plot, both types of H-bond can smoothly interconvert during bending without having to cross large energy barriers.^79^ To date, the functional cycles of only a few other membrane proteins are documented by high-resolution structures as extensively as with Ca^2+^-ATPase. Nonetheless, analyzing a broader database of membrane proteins identified bifurcated H-bonds, where (i,i-3) and (i,i-4) binding occurs in parallel.^80^ Provided that bifurcation in a given state is indicative of potential interconversion, this finding may hint at a widespread mechanism by which TM-helices can bend. Bifurcated intrahelical H-bonds of this type are frequent in globular proteins, too.^12^

By comparison to the α-to-3_10_ switch, the more rarely occurring π–helical turns are energetically more unfavorable.^81^ Specifically, the low abundance of π-helices results from their main-chain torsional angles being less favorable relative to the α-helix, their backbone being less stabilized by van der Waals’ interactions, and its formation being kinetically disfavored as four residues must be correctly oriented before the first (i,i-5) H-bond can form.^13^ The conformational flexibility of the different types of helixes has been compared by *B*-factor analysis. *B*-Factors describe the extent to which X-ray diffraction by atoms in a high-resolution crystal structure is attenuated by local disorder. Upon normalization to eliminate confounding influences, such as disorder in the crystal lattice, *B*-factors reflect thermal motions and thus reflect sites of conformational flexibility.^82^ In globular proteins, the normalized *B*-factors follow a rank order of 3_10_-helix (*B*-factor = 0.24) > π-helix (0.09) > α-helix (−0.12). The *B*-factors of the different helix types correlated reasonably well with the respective root-mean-square fluctuations of atoms determined by molecular dynamics (MD) simulations, a measure of local structural fluctuations.^83^ In sum, local 3_10_-helices and π-helices should be regarded as potential hotspots of flexibility.

Another way of predicting protein dynamics is to assess the density of the packing within their cores. Interestingly, Adamian and Liang found that membrane proteins are on average less densely packed than soluble proteins suggesting their generally higher flexibility.^84^ In agreement with the low packing density, significant side-chain flexibility has recently been observed for one helical membrane protein, the bacterial sensory rhodopsin II that plays a role in phototaxis by activating a transducer protein. In this case, NMR spectroscopy unveiled interconversions between different side-chain rotameric states at the subnanosecond time scale. Motions at this time scale are virtually absent in most soluble proteins.^85^ Channels and transporters are even more loosely packed than other multipass membrane proteins as shown by Hildebrand et al. Loose packing results in a greater amount of cavities which are particularly prevalent at pores or the hinges mediating the gating movements.^86^ Further, analyzing the geometries of 3D-structures revealed a larger overall variance of backbone torsion angles across, as well as their deviation (Δφ = +3,8°, Δψ = −3.6°) from, the TM helices of channels and transporters relative to other membrane proteins.^80^ In addition, the membrane cores of channels and transporters frequently contain coil regions. The coil regions are usually more evolutionarily conserved than noncoil regions which attests to their functional importance. One function of coils may relate to the transient binding of polar transported substrates and another one to the large conformational changes required for gating. A polar unfolded polypeptide chain and polar side chains of deep-membrane coils mostly H-bond to the protein when buried and/or are exposed to aqueous pores.^52^

### 2.3 Interconversion between Functional Substates of Bacteriorhodopsin Involves TM-Helix Dynamics

While a myriad of membrane protein structures has been solved within the five decades since Unwin and Henderson’s first structure of bR, bR and various other rhodopsins continue to provide fresh insights into the structural dynamics during a functional cycle. Within the limited database of known membrane protein structures known in 2001, Adamian and Liang detected the highest packing density within a high-resolution bR structure.^84^ Despite dense packing, comparing the key structural substates of bR unveils moderate changes in TM-helix bending angles along its functional cycle, as briefly described in the following.

bR is a light-driven proton pump with seven TM-helices (A–G) and a retinal chromophore covalently attached to helix G via a Schiff base (Figure 2A). The imminent question is how absorption of light is translated into the transport of a proton from the cytoplasm to the extracellular space, thus contributing to the proton gradient driving bacterial ATP production by ATP synthase (reviewed in ref 87). It has long been known that starting with the dark state the photocycle of bR consists of spectroscopically distinct I, J, K, L, M, N, and O intermediates and is completed within ∼15 ms. Having been elucidated in ever increasing detail by decades of research, the underlying series of molecular events has more lately been studied on time scales from femtoseconds to milliseconds at room temperature using time-resolved crystallography.^87,88^ For data collection, crystals within a continuous stream of liquid pass short pulses of light before being analyzed by X-ray diffraction after different periods of time. It thus has been possible to characterize structural intermediates that are associated with distinct functional states. Mechanistically, the photocycle can roughly be subdivided into two processes: steps leading to proton release at the extracellular space and steps in which bR is recharged with a proton from the cytoplasm. In the first process, bR is activated by photoisomerization of the all-*trans* retinal to the 13-*cis* conformation. From the resting or dark state, substates I to K are reached within femtoseconds to picoseconds and correspond to structural intermediates of retinal isomerization.^89^ This elicits subtle conformational changes within the protein that alter the local polarity. In the ensuing L-to-M state transition, altered polarity causes the transfer of a proton from the Schiff base to residue D85 as a result of their transiently altered acidities. Other ionizable residues and interspersed water molecules create a pathway along which the liberated proton then moves unidirectionally toward the extracellular space. A structure developing within a few milliseconds is believed to be the equivalent of the M-state. The structural changes from dark-state to M-state mainly progress from helix G, were retinal is attached, to helices F and C.^87,89–91^ In the dark state, helix C exhibits a kink a few residues upstream of a Pro residue (P91). P91 is highly conserved in bR.^92^ Similar to the other highly conserved P186 (helix F) and the semiconserved P50 (helix B), P91 has proven to be essential for bR structure and function.^92^ Specifically, P91 and its vicinity are important for signal transduction from the retinal to the rest of the protein and its mutation slows down protein dynamics.^93^ Once the M-state is reached, helix C flexes by ∼1 Å toward helix G (Figure 2B). The second process leads to the reprotonation of the Schiff base by a proton delivered from the cytoplasm. The corresponding N-state is captured by a structure mainly developing from 10 to 15 ms.^91^ In the M-state, L93 and the neighboring F219 concomitantly change their rotameric states, thus permitting the formation of a water wire along which a proton enters from the cytoplasm to reprotonate the Schiff base for the next pumping cycle. During the M- to N-state transition, helices E and F flex outward by ∼9 Å at their termini in a movement which is associated with bending at their respective kinks. The kink of helix E is located near a Gly residue (G155). Altogether, the functional cycle of bR is composed of concerted structural changes: isomerization of the chromophore structure, local polarity changes within the protein that result in altered proton affinities, TM-helix bending, and side-chain rotation (see the instructive movie S2 in ref 91).

**Figure 2:**
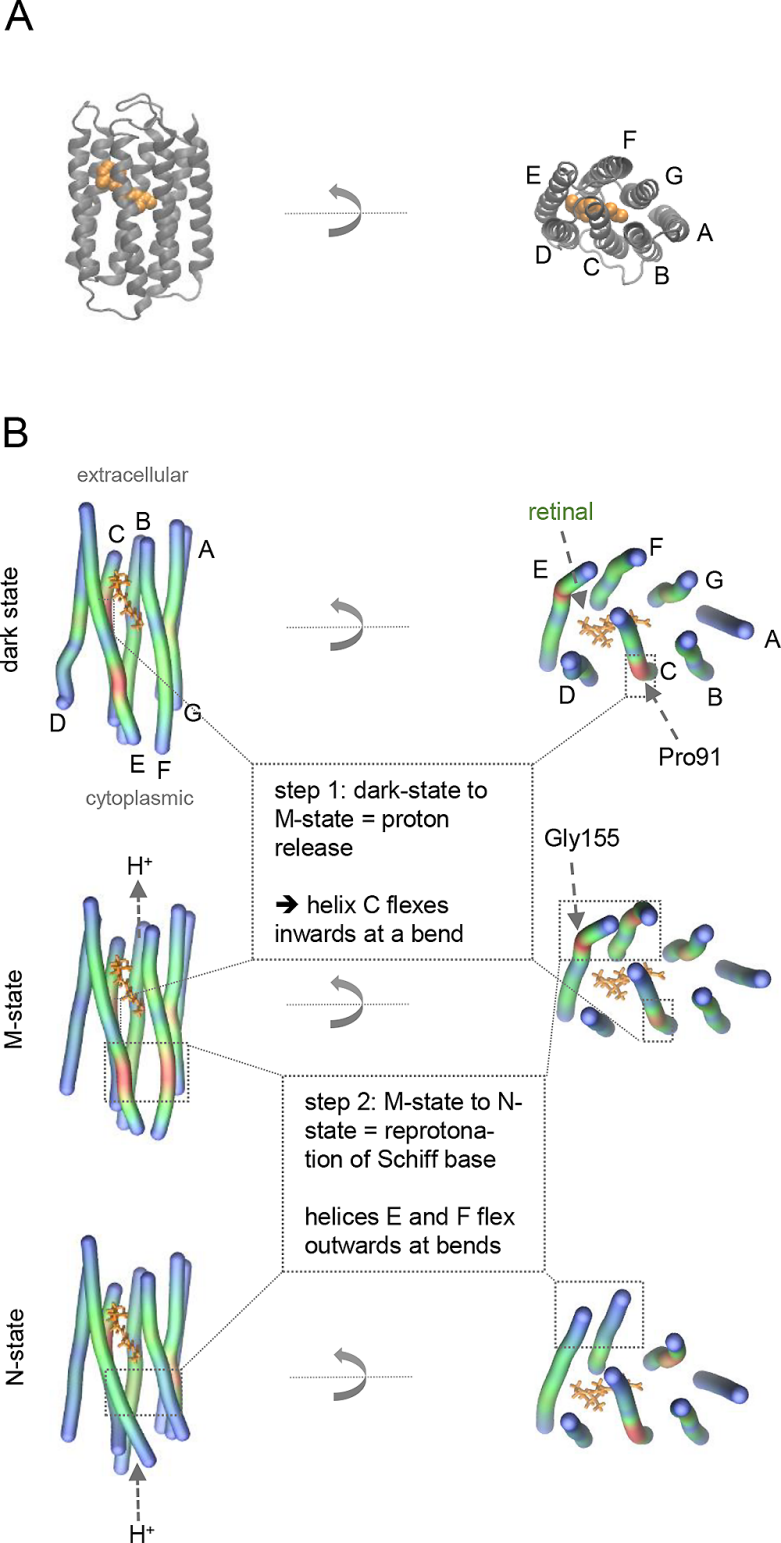
Structure of bR as a paradigm of how crude models can evolve into multiple high-resolution structures that reveal a connection between functionality and TM-helix dynamics. (A) bR model at atomic resolution (pdb 2BRD^94^) that was obtained 20 years after the initial plywood model.^1^ (B) Crucial conformations with a focus on TM helix kinks along the bR photocycle as identified by time-resolved crystallography. Top, dark state (6G7K^90^); center, putative M-state (6RNJ^91^); lower, putative N-state (instead of 6RPH, that was obtained by time-resolved crystallography,^91^ but could not be evaluated by BENDIX, the very similar 4FPD structure^95^ was chosen for representation). The BENDIX plugin^96^ within VMD^97^ was used here to illustrate the bending angles that increase from blue to red (spine setting = 4, side setting = 7.2). The retinal chromophore in (B) and (C) is represented in orange.

Significant conformational changes also accompany the functional cycle of the visual pigment rhodopsin. Although unrelated in sequence and structure to bR and other bacterial rhodopsins,^48^ rhodopsin also contains a retinal, whose light-induced *cis/tran*s isomerization eventually results in binding and activation of transducin, a trimeric G-protein, with further consequences ultimately defining the electrical activity of retinal cells. Some of rhodopsin’s TM-helices are bent near Pro residues and contain non-α-helical elements. Altered spatial constraints on the TM-helices upon chromophore isomerization alters helix bending angles and rearranges local H-bonding patterns that ultimately increase the affinity for transducin at its cytoplasmic side (reviewed by refs 98–100 ). Since rhodopsin is structurally related to other G-protein-coupled receptors that share its 7-TM-helix topology, insights into its mode of function are of great relevance for understanding how the binding of their respective ligands impacts the functional cycles of many important drug targets.^101^

### 2.4 Ion Channel Gating Involves Changes in TM-Helix Packing and Flexibility

The most loosely packed membrane protein found in the aforementioned analysis in 2001 ^84^ corresponds to the mechanosensitive channel of large conductance (MscL). Residing within a bacterial membrane, MscL opens in response to decreasing osmotic pressure in the environment (reviewed in ref 102). Low osmolarity triggers a flow of water into the cell, thus causing cell swelling and a reduced lateral membrane pressure in the process. The channel is a homopentamer of subunits with two TM-helices, each (TM1 and TM2) with a cytoplasmic domain. TM1 is preceded by a short N-helix. Its structures in open and closed states have been solved by X-ray crystallography,^103^ which revealed that TM1 helices form antiparallel pairs with TM2 helices from neighboring subunits (Figure 3). In the closed state, TM1 helices form a hydrophobic constriction in the center of the pore. Decreasing lateral pressure leads to strong tilting of the TM1–TM2 pairs, which is associated with massive rearrangements of the TM-helical bundle resulting in pore opening. While the intersubunit TM1–TM2 pairs move together largely as rigid bodies, tilting also involves a redefinition of the weak TM1–TM1 contacts. In addition, a kink at E58 near the periplasmic end of TM1 disappears upon helix tilting. This disappearance results from the unfolding of its C-terminal region, which includes E58. At the same time, gating causes asymmetric conformational changes in the molecule. The compressed open state exhibits a strong kink within TM2 of subunit D (and E) at a triple Ile motif (I99–I101) while the TM2 helices of subunits A–C avoid kinking by getting shorter at their N-terminal end. Lastly, channel-forming TM1 develops a slight kink at G46. The diameter of the channel in the highly dynamic open state^104^ is ∼30 Å, which allows for the discharging of cytoplasmic solutes, thus rescuing the bacterial cell from rupture. The gating of some mammalian mechanosensitive channel proteins might depend on surrounding membrane tension in similar ways.^105^

**Figure 3:**
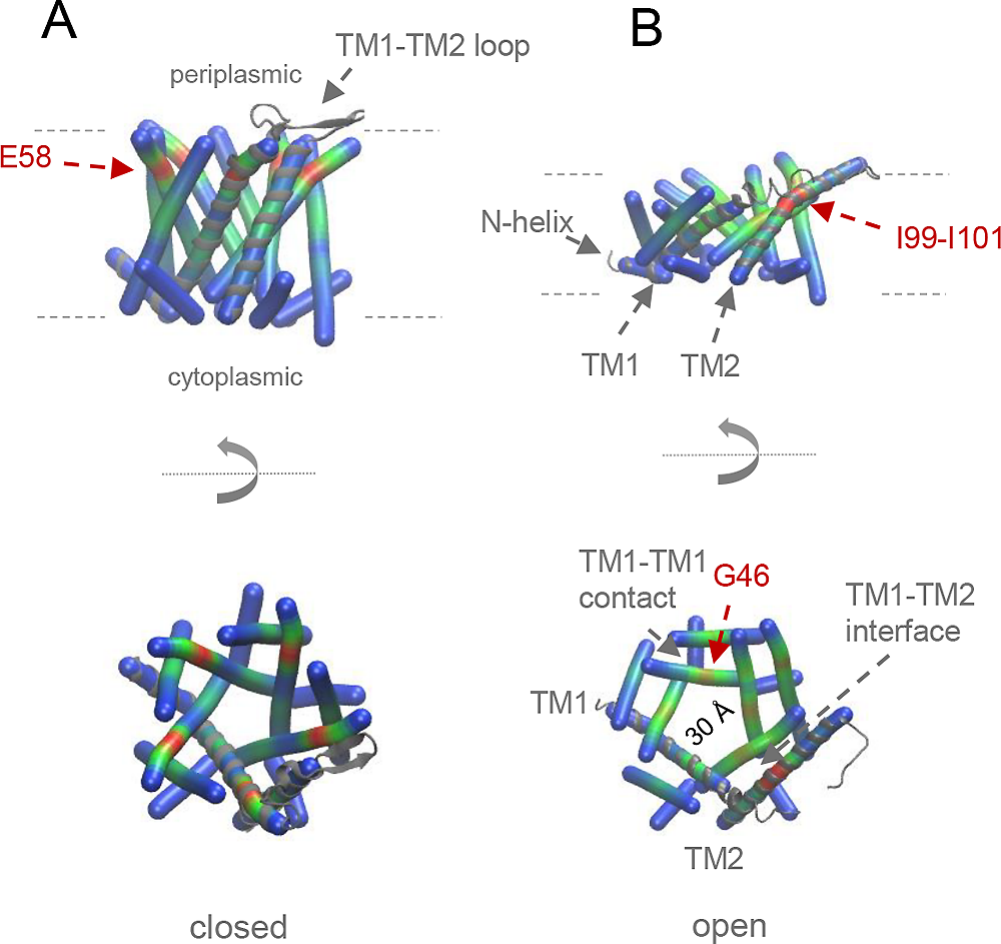
Massive structural changes are associated with MscL channel gating. (A) Closed (pdb 4Y7K) and (B) open (pdb 4Y7J) structures^103^ of the MscL homopentamer in BENDIX representation to reveal changes in helix bending. Upper panels: Side view of the membrane-spanning parts of the channel. Bottom panels: top view as viewed from the periplasmic side. For better orientation, subunit D is marked with a gray ribbon. In the closed state, TM1 helices are five to nine residues longer at their C-terminal end than in the open state. This is paralleled by kink formation at E58 near the lipid headgroup region. Pore opening is associated with a massive reorganization of the helical bundle which results in a severe compression of the molecule in a thinner surrounding lipid bilayer (symbolized by broken lines). Specifically, gating involves stronger tilting of both TM-helices, a larger angle between the N-helix and TM1, and a redefinition of the TM1–TM2 contacts. In addition, TM2 of subunits A through C shorten by ∼10 residues at their N-terminal ends. TM2 of both other subunits essentially remain at their original lengths causing a kink at an Ile triplet (I99–I101) within TM2 of subunit D and to a lesser extent within TM2 of subunit E. Moreover, TM1 develops a slight kink at G46. The loops between the periplasmic termini of both helices act as springs that modulate gating. See the caption of Figure 1 for BENDIX usage.

By contrast to MscL, the structural changes associated with the gating of ligand-gated ion channels are rather small. Ligand-gated ion channels translate agonist binding to the opening of a pore being selective for certain cations or anions. They form a superfamily of pentameric proteins, including neurotransmitter receptors for acetylcholine, serotonin, γ-aminobutyric acid, and glycine. The nicotinic acetylcholine receptor (nAChR) regulates nerve-muscle transmission at the neuromuscular junction in vertebrates where it exists as a heteropentamer with the arrangement of α_γ_, γ, α_δ_, δ, and β subunits (reviewed in ref 106). Electrophysiology showed that channel gating involves rapid open–close transitions on a submillisecond time scale. Thus, the receptor interconverts rapidly between three major functional states, the closed resting state and the open state, plus a desensitized closed state that follows the open state within tens of milliseconds. Although the time spent in these states depends on the presence of agonists or antagonists, they appear to exist independently of the ligand, as demonstrated by spontaneous channel openings. Different functional conformations thus appear to be in equilibrium with each other rather than being triggered by ligand binding. Like all ligand-gated ion channel proteins, the nAChR subunits have four TM-helices (M1–M4) where a pentameric assembly of M2 helices forms the central ion pore. Research has mainly focused on the mechanism explaining how agonist binding favors the open state and how the functional cycle is modulated by allosteric ligands and lipids. Due to its abundance in the electric organ of the electric ray, a muscle-derived tissue, the muscle nAChR has become an early paradigm of ligand-gated ion channels and its structure was thus the first to be elucidated by Unwin and co-workers at atomic resolution by cryo electron microscopy (cryo-EM).^107^ In accord with the closed states determined later for other ligand-gated ion channels, the ion pore is obstructed by a central constriction formed by bulky hydrophobic residues halfway down the channel-forming M2 helix. Structures in the presence of an agonist revealed that occupation of an agonist binding site at the interface between the soluble domains of α and γ subunits translates into a force that pulls on the M2–M3 linker. An outward displacement of the linker and its adjoining TMDs leads to tilting of M2 helices relative to each other and to the membrane normal, as well as rotation of the constricting residue side-chains. In this state, however, the pore is still too narrow for ion passage, suggesting that it already represents the nonconducting desensitized state.^108,109^ In a time-resolved approach, the receptor from *Torpedo* was therefore freeze-trapped in the open state within a few milliseconds of agonist application in order to prevent the desensitized state to build-up. Electron microscopy subsequently found that agonist binding causes the relaxation of the M2 helices of α and δ subunits from a slightly curved to a straight state (Figure 4).^110^ Although this change in M2 conformation is rather subtle, it is thought to allow for the rapid flexing of the M2 helices that leads to the disappearance of the central constriction required for repetitive open/close transitions. A more recent study also suggested a modulatory impact of intrasubunit TM-helix–helix packing on channel gating. Specifically, the embryonic isoform of the mammalian muscle-type nAChR contains a γ-subunit, which confers a number of functional characteristics that differ from the adult receptor where γ is replaced by ε. For example, the γV18/εI18 substitution within M2 was speculated to increase the single channel conductance by stabilizing the helix through interactions with residues of surrounding helices. Similarly, an electrostatic interaction network mediated by γT316 (M3) between M3, M2, and M1 as well as a hydrophobic network mediated by γM482 (M4) that inserts into a pocket formed by residues from M1, M3, and M4 have been proposed to regulate channel open time by stabilizing the helical bundle.^109^ Taken together, the equilibrium between closed and open ligand-gated ion channel states is affected by multiple influences: the agonist-dependent force exerted by the extracellular domains on the TM-helix bundle as well as the packing interactions within that bundle. Both effects appear to affect the probability of channel opening and its conductance via conformational changes in the pore-lining ensemble of M2 helices. M2 conformations thus crucially depend on its packing interactions with the surrounding TM helices.

**Figure 4:**
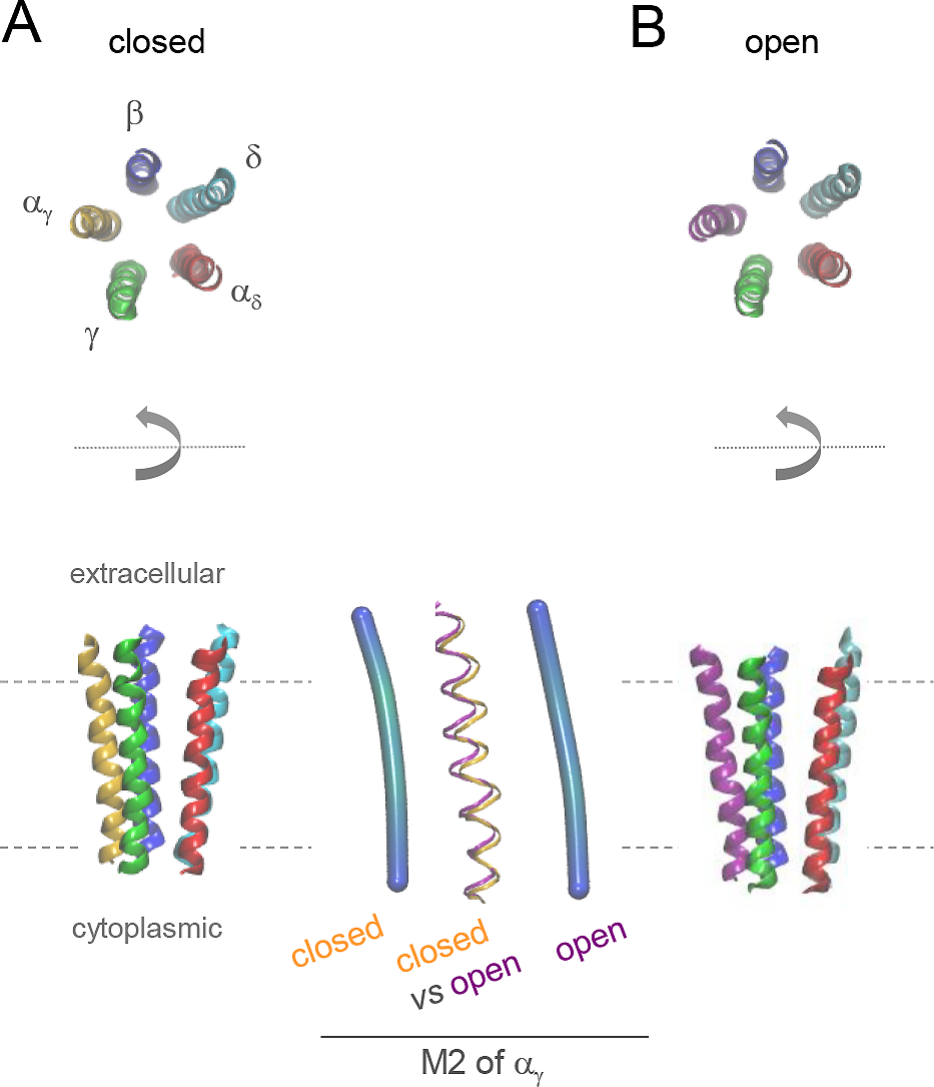
Subtle structural changes upon ligand-gated ion channel gating. The heteropentameric assembly of channel-forming M2 helices from the *Torpedo marmorata* nAChR in the closed (A) and open (B) state.^110^ All other receptor domains are hidden for the sake of clarity. Upper panel: Top view of the channel viewed from the extracellular side. Lower panel: Side view. The center of the lower panel shows an enlarged overlay of the M2 helices of the α_δ_ subunit in both states which reveals that M2 straightens somewhat upon gating. BENDIX confirms the slightly stronger curvature of M2 in the closed (pdb: 4AQ5) state compared with the open (4AQ9) state of the receptor. See the caption of Figure 2 for BENDIX usage.

In summary, different deviations from canonical α-helicity in 3D structures often mark sites of conformational flexibility. The tolerance for Pro within the core of TM-helices makes membrane proteins apparently use this amino acid to induce hinges, while the stabilizing influence of the lipid bilayer prevents complete helix unfolding. Hinges formed near Pro and, to a lesser extent, near Ser, Thr (and Cys) permit functionally relevant bending and swivel motions^111^ as demonstrated by a diversity of ion pumps and channels where multiple structural substates are known.

## 3 The Conformational Flexibility of TM-Helices of Single-Span Membrane Proteins

Here, we look into the use of model peptides in assessing the relationship between helix dynamics and the primary structure. In doing that, the earlier sections of this section will confirm and complement sections 2.1 and 2.2. Also, we will see how the polarity of the environment as well as the methodological approaches used influence the conclusions drawn, which has interesting theoretical ramifications. Applications of the findings include the mechanism of lipid mixing as well as substrate cleavage by intramembrane proteases.

### 3.1 Host–Guest Studies Report on the Stability of Model Helices

Host–guest studies have established the impact of various guest amino acids on the conformation of invariant host peptides. In the earlier studies, circular dichroism (CD) spectroscopy was mostly used to probe the helicity in different solvents. The magnitude of CD depends on differential absorption of the left-handed and right-handed components of circularly polarized light by chiral secondary structure in the far-UV. A changing CD is thus sensitive to drastic conformational changes such as helix unfolding. In host–guest studies, CD therefore reports the extent to which introducing a given guest amino acid shifts the helix/coil equilibrium. In soluble peptides dissolved in aqueous media, Ala turned out to be the most helix-stabilizing amino acid and Pro the most destabilizing one (refs 28–31 as reviewed in ref 13). Pro also proved to be most disruptive amino acid in mutational studies done with globular model proteins.^9,27^ The following rank order of helix-unfolding power in aqueous solution, restricted to the limited set of crucial amino acids discussed in the scope of the present review, was obtained: Pro ≫ Gly > Thr ≈ Val ≥ Ser ≥ Ile > Leu, while Ala was regarded as the most helix-stabilizing residue (Table 2).^32^

**Table 2:** Rank Order of Helix-Stabilizing Amino Acid Types As Found in Host–Guest Studies

evidence	order of helix-destabilization	most helix-stabilizing	ref
unfolding in aqueous solution probed by CD spectroscopy	Pro ≫ Gly > Thr ≈ Val ≥ Ser ≥ Ile > Leu	Ala	^9^,^28–32^,^113^
unfolding in apolar environments probed by CD spectroscopy	Pro ≫ Gly > Ser ≈ Thr > Ala	Val ≈ Ile ≈ Leu	^30^,^31^
backbone fluctuations in TFE/water probed by DHX and MD simulation	Gly > Ala > Val	Leu	^22^,^23^,^36^,^114^,^115^

The host–guest approach was adopted by Deber and co-workers to probe the impact of various guest amino acids on the folding equilibria of hydrophobic host sequences in apolar environments, again assessed by CD spectroscopy. In agreement with the work done in aqueous solution, Pro residues had the strongest destabilizing effect of all amino acids on host peptides in alcohols, detergent, or lysolipid micelles, as well as in liposomal membranes.^30,31^ Clearly, however, stronger H-bonding within these apolar environments^71,72^ prevented Pro to cause the virtually complete helix unfolding seen in aqueous solution. Compared to Pro, multiple Gly guests caused a much smaller shift in the helix/coil equilibrium. Ser and Thr also had intermediate impacts on helix stability being comparable to those of Gly. Overall, the helix-destabilizing power, as determined by the host–guest approach in apolar environments, roughly followed the rank order Pro≫Gly > Ser ≈ Thr > Ala with Val, Ile, and Leu at the most helix-stabilizing end.^30,31^ Considering that the β-branched Val and Ile tended to be helix-destabilizing in aqueous solution (rank order: Val > Ile > Leu), as noted above,^13,30,32^ the apparently near uniform helix-promoting effect of the aliphatic residues (Val ≈ Ile ≈ Leu) in hydrophobic environments was an unexpected outcome.^30,31^

A structured framework, “AAontology”, recently classified hundreds of published physicochemical amino acid scales obtained from many different proteins in different environments. This classification reflects the spread of the underlying data and confirms a slightly more helix-stabilizing function of Leu, compared to Ile and Val.^112^

### 3.2 The Impact of Val on Helix Flexibility As Measured by Deuterium/Hydrogen Exchange

Intrigued by this discrepancy, we investigated the potential impact of Val on helix flexibility using a different approach. Specifically, we probed the impact of Val on the vibrational backbone motions of a host helix, giving rise to transient amide H-bond openings rather than unfolding. This was initially motivated by a conspicuous overabundance of Ile and Val in the TMDs of membrane–fusogenic proteins, hinting at their functional relevance.^116^

One way to monitor backbone fluctuations is hydrogen/deuterium-exchange experiments. This method monitors transient openings of main-chain amide H-bonds that permit successive exchange of hydrogens for deuterons (HDX) or *vice versa* (DHX), depending on the experimental design. Exchange rates thus provide a measure of conformational flexibility along a helix.^117–119^ The kinetics of amide exchange can be determined by NMR spectroscopy,^119^ Fourier transform infrared spectroscopy,^120^ or electrospray ionization mass spectrometry (ESI-MS).^121^ While NMR spectroscopy detects the disappearance of the paramagnetic hydrogen signals, infrared spectroscopy relies on the different frequencies of stretching vibrations of N–H vs N–D bonds and ESI-MS measures the differential molecular masses of protiated and deuterated peptides. The advantages of using ESI-MS include that low micromolar sample concentrations prevent aggregation and that the speed of measurement allows recording very early time points of the exchange kinetics. As my lab has gained considerable experience in investigating synthetic transmembrane helices using the ESI-MS approach, I will provide a brief account of its potentials and limitations. Theoretical and experimental details are given in the captions of Figures 5 and 8.

**Figure 5:**
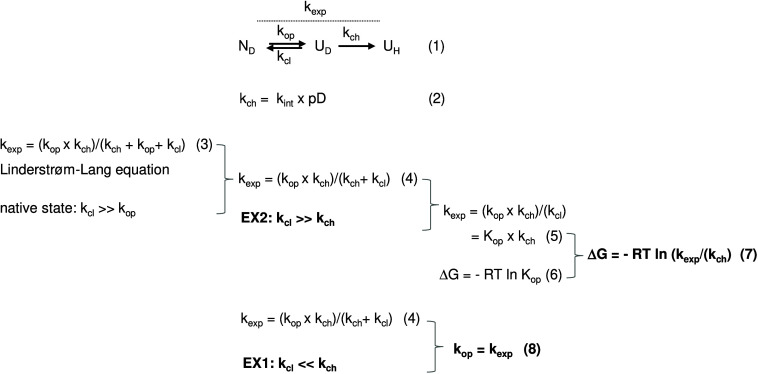
Interpretation of DHX kinetics. According to eq 1, local or global unfolding from a deuterated native state *N* of a helix to an unfolded state *U* proceeds with the open rate constant *k*_op_; closing is described by the rate constant *k*_cl_. From *U*, amide exchange can occur with the exchange rate constant *k*_ch_; *k*_ch_ is the product of the intrinsic exchange rate constant *k*_int_ and hydroxide ion concentration given by the pD or pH value (eq 2). At pD or pH values ≥4, the exchange chemistry is essentially base-catalyzed. Values of *k*_int_ depend on local sequence context, i.e., inductive and steric effects of neighboring side chains and were calculated based on model peptide studies for HDX^125^ or DHX,^126^ respectively. The calculation of *k*_ch_ can be done using tools developed by the Englander lab (https://hx2.med.upenn.edu/download.html). Exchange is practically irreversible if D_2_O or H_2_O, respectively, are present at a vast excess in the sample. The Linderstrøm–Lang eq 3 captures the dependence of the experimentally accessible *k*_exp_ on *k*_op_, *k*_cl_, and *k*_ch_. Under the assumption that a structure exists mainly in the *N* state, where *k*_cl_ ≫ *k*_op_, eq 3 simplifies to eq 4. Exchange operates under the EX2 regime if *k*_cl_ ≫ *k*_ch_, where many open–close reactions occur before the rare exchange events can happen. EX2 exchange reactions are thus uncorrelated. In this case, eq 4 simplifies to eq 5 where *k*_exp_ relates linearly to the stability constant *K*_op_! The Gibbs free energy Δ*G* associated with the opening of a given amide H-bond, as resulting from local unfolding, is readily calculated from *K*_op_ (eq 6), i.e., it can be derived from *k*_exp_ and *k*_ch_ (eq 7). In the case that *k*_ch_ ≫ *k*_cl_, exchange operates under the EX1 regime where exchange reactions are correlated. There, eq 4 simplifies to eq 8 where the kinetics of H-bond opening *k*_op_ equals *k*_exp_ (see also Figures 6 and 7).

In essence, labile hydrogens bound to amide nitrogens of a protein’s main chain can exchange unless they are protected by H-bonding and/or burial within the folded core of the protein. Burial is irrelevant in a small helical peptide, where exchange consequently reflects local main-chain fluctuations and/or its wholesale unfolding into the coil state. The experimentally measurable exchange kinetics are described by the Linderstrøm-Lang eq (Figure 5). At pH 4 and above, exchange is mainly catalyzed by hydroxide ions, and its kinetics is thus linearly related to their concentration. The velocity of exchange is thus set by the pH (DHX) or pD (HDX) value. Investigating backbone fluctuations of a helix requires that exchange be carried out in the EX2 regime. In this case, exchange is slower than refolding such that transient amide H-bond openings only allow for rare and thus uncorrelated exchange reactions (Figures 5 and 6). One way to interpret the resulting gradual decrease in time of a peptide’s mass is to determine its *overall* exchange kinetics, i.e., looking at the complete peptide. Overall kinetics can mathematically be deconvoluted into exchange rate constants *k*_exp,*i*_ to *k*_exp,*y*_ that reflect the probabilities of H-bond opening in different parts of a helix. Higher *k*_exp_ usually correspond to frayed termini and/or flexible hinges while lower *k*_exp_ describe more rigid central parts. By contrast to EX2, correlated exchange in the EX1 regime is promoted by long-lived open states that can correspond to partially unfolded helices or a random coil. EX1 is also promoted by very high hydroxide concentrations. In its pure form, EX1 kinetics can yield the rate constant *k*_op_ of unfolding. In practice, EX2 kinetics are often mixed with an EX1 component, as indicated by intermittent peak broadening during predominantly uncorrelated DHX.^118,122–124^

**Figure 6:**
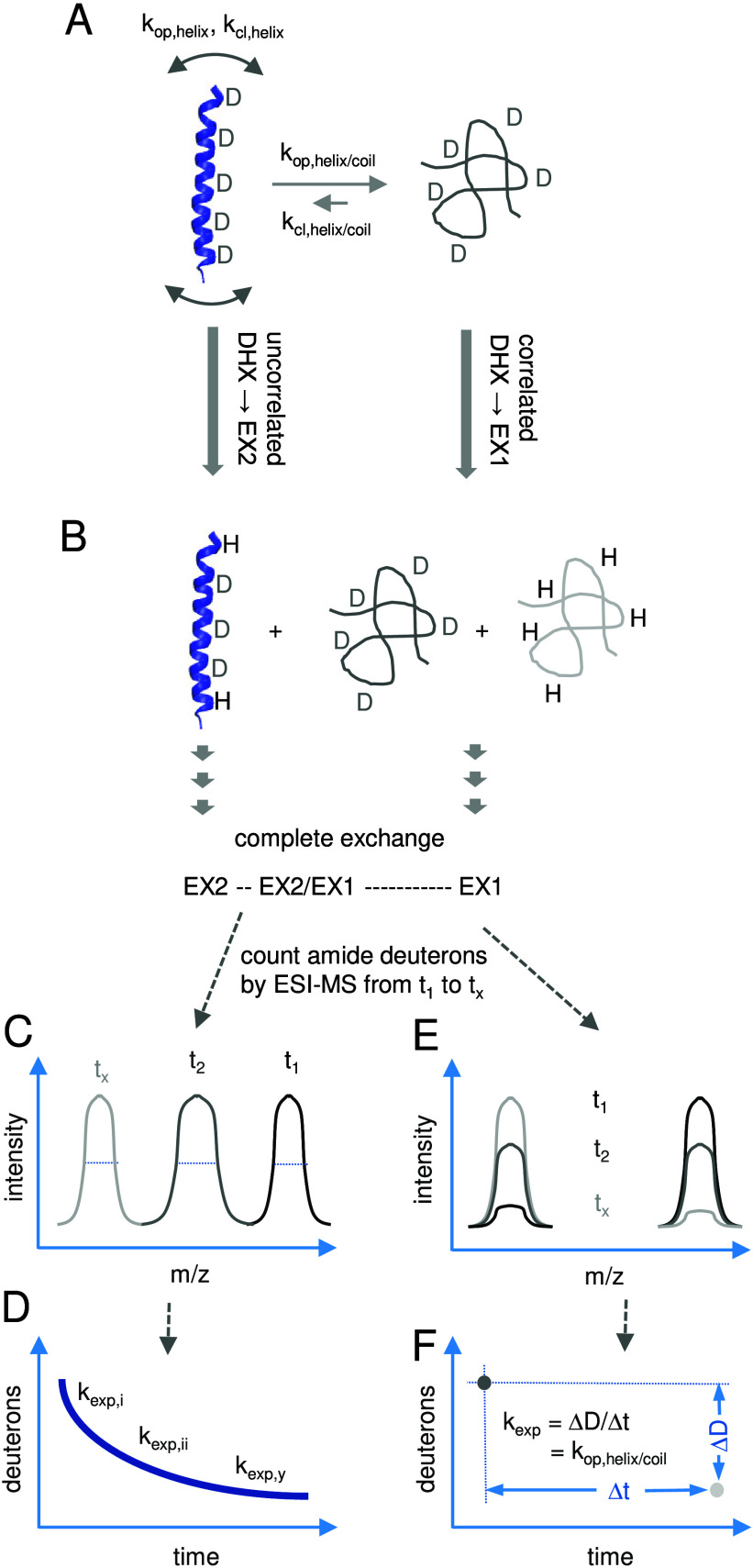
Describing the conformational flexibility of a helix by its *overall* DHX kinetics. (A) A TMD peptide to be investigated is depicted that exists predominantly as a conformationally flexible helix (blue) being in equilibrium with a minor coil state (green). On the one hand, limited local conformational fluctuations (symbolized by curved double arrows) along the helix are characterized by a range of corresponding rate constants of *local* unfolding (*k*_op,helix_) and folding (*k*_cl,helix_). On the other hand, the kinetics of the helix/coil transition is described by the rate constants of *global* unfolding (*k*_op,helix/coil_) and folding (*k*_cl,helix/coil_). (B) Uncorrelated DHX (left) in the EX2 regime of the exhaustively deuterated flexible helix is induced by adding a surplus of protiated solvent. Exchange starts at helix termini before proceeding to the core, given that the frayed termini represent the most flexible parts of the helix. Correlated DHX in the EX1 regime is observed at unfolded regions or conformations that exist long enough for multiple exchange reactions to occur before refolding occurs (see: Figure 5). In case of a coil state, correlated DHX can thus completely protiate increasing fractions of the molecules (in light green, right). Experimentally, DHX is continued until near complete amide exchange in both cases. (C) A purely uncorrelated EX2-type of DHX leads to isotopic envelopes that gradually shift to lower *m*/*z* values, as determined by ESI-MS at discrete time points *t*_1_ to *t*_*x*_ (or online). The *m*/*z* values are subjected to a number of corrections (see caption of Figure 7). Plotting the numbers of remaining deuterons, as obtained from average masses, over time yields the decay curve (D) which may be fitted with multiple exponentials^121,127^ or with a maximum entropy method^128^ to obtain *k*_exp,*i*_ to *k*_exp,*y*_, along with the respective populations of deuterons. Higher values of *k*_exp_ are likely to reflect groups of more quickly exchanging amides at helix termini while lower values result from slower exchange at more stable helix cores. (E) A purely correlated EX1-type of DHX results in bimodal mass spectra where the decreasing prevalence of the fully deuterated state is gradually compensated for by the increasing prevalence of the fully protiated one. As depicted in (F), *k*_exp_ determined from purely bimodal exchange corresponds to the rate constant of helix unfolding *k*_op_ (see: Figure 5). In practice, mixed EX2/EX1-type of DHX are often observed where exchange at a major folded region of a peptide is largely uncorrelated while minor unfolded regions exchange in a correlated fashion.^129^ In these mixed cases, a contribution of EX1 exchange is indicated by broadening of the isotopic envelope at intermediate time points,^130^ as hinted by the broader isotopic envelope in the center of part (C).

As outlined above, studying the helix/coil equilibria of host–guest peptides in polar solvent had indicated a helix-destabilizing function of the β–branched Val and Ile, relative to Leu. By contrast, experiments done in apolar media had suggested that these aliphatic amino acids contribute similarly to helix stabilization (Table 2). To resolve the issue, we designed sequences with increasing Val/Leu ratios, termed LV-peptides. One derivative also holds a GlyPro pair to explore the limits of flexibility (Figure 7 A). Unfortunately, membrane-embedded parts of a helix are not accessible to the catalytic hydroxide ions and thus experience only limited exchange.^36,127,131,132^ Therefore, we used 80% trifluorethanol (TFE) in buffered aqueous solution (TFE/water) as a solvent (see NOTES on DHX-MS in the caption of Figure 7 for the virtues of using TFE). In TFE/water, CD spectroscopy reported only a very limited impact of the Val content on helicity. By contrast, an increased Val/Leu ratio strongly affected DHX kinetics (Figure 7B–E). MD simulations confirmed the impact of Val on helix flexibility and showed that the structural fluctuations of LV-helices increase from their cores toward the frayed termini. As expected, the GlyPro pair accelerated DHX further which is ascribed to kink formation (Figure 7F).^23,36^ In TFE/water, poly-Val mainly formed a β-sheet^114^ although MD simulation suggested formation of a highly flexible helix by an oligoVal sequence^133^ that might be stable in the absence of sheet-forming partners. What is the mechanism by which Val enhances backbone fluctuations of a helix? For sterical reasons, only one out of three possible rotamers of the Val side chain is populated in a helix.^25,26,38^ Resulting from this conformational restriction and its relatively small size, the Val side chain makes less favorable intrahelical contacts compared to Leu with its larger and less restricted side chain (Figures 2F and 7G,H). In the simulation, side-chain/side-chain interactions proved to be much more crucial for flexibility than side-chain/main-chain contacts.^23^ This finding aligns well with an earlier concept were the helix propensity of residues corresponds to their respective buried hydrophobic areas upon helix formation.^27,35^ In TFE/water, burial of hydrophobic area mainly translates into intrahelical van der Waals interactions^23^ as the hydrophobic effect is likely to be of limited relevance. The reduced intrahelical packing interactions of Val are expected to accelerate helix unfolding relative to Leu, a prediction confirmed by kinetic measurements of another helical model system where Val indeed caused faster local helix unfolding than Leu.^40^ The thermodynamic scheme in Figure 1G summarizes the contributions to the free energy of a helix. It is likely that the enthalphic contributions made by side-chain interactions dominate the entropic contributions to the free energy of a helix, provided that the helix mainly experiences local structural fluctuations, rather than complete unfolding. See the NOTES in the caption of Figure 1 for an expanded discussion of this issue.

**Figure 7:**
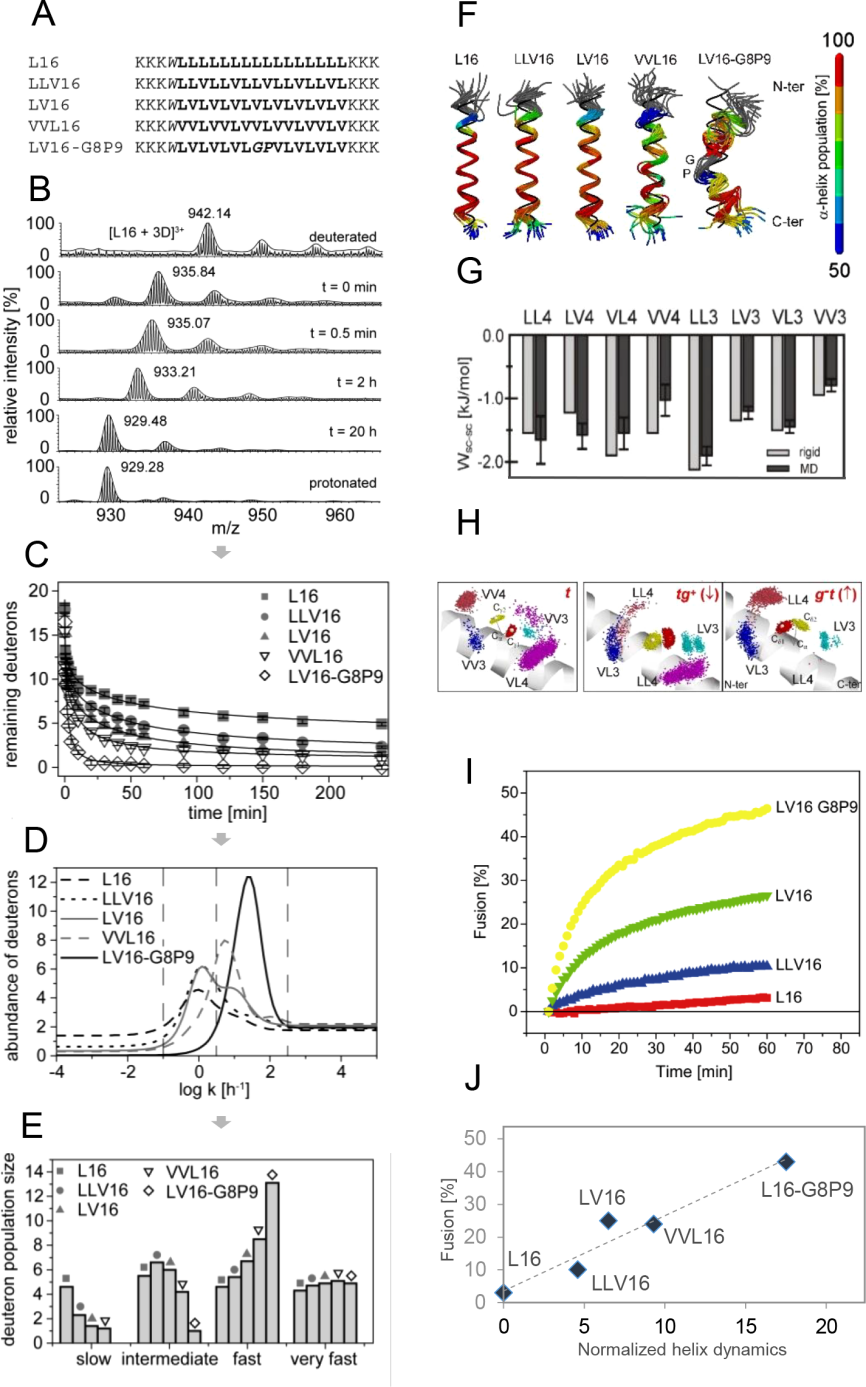
Impact of Val on the conformational flexibility of model helices. (A) Sequences of LV-peptides. Invariant Lys tripletts render the peptides soluble and prevent aggregation. (B) Exemplary mass spectra of the triply charged ion of the L16 host sequence after different periods of DHX reveal the gradual shift of the isotopic envelopes toward lower masses, which is diagnostic of EX2 kinetics. (C) Exchange kinetics were fitted using a maximum entropy method to yield the sequence-specific distributions of rate constants shown in (D). (E) Kinetically distinct populations of amide deuterons, as defined by different ranges of *k*_exp_ in part (D), were grouped into four different categories that collectively characterize the conformational flexibility of the peptides. CD spectroscopy revealed comparable helicities of the different peptides. (F) MD simulations confirm that backbone fluctuations of LV-helices increase with Val content and the introduction of a GlyPro pair. (G) Computed average van der Waals interaction energies (*W*_sc‑sc_) between side-chain pairs at (i,i ± 3,4) spacing in LV-peptides. Averages from MD trajectories (black) are compared to averages calculated for residue pairs of a rigid α-helix (gray). (H) Graphical depiction of transient contacts by dots. The dots represent van der Waals contacts seen during the simulations. This is illustrated for residue V12 which populates mainly the *trans* rotamer in peptide VVL16 (left panel, reproduced from Figure 1 F) and for residue L13 that swaps between *tg* ^*+*^ (center panel) and *g* ^*‑*^ *t* in peptide LV16 (right panel). (I) The extents of liposome–liposome fusion induced by membrane-embedded LV-peptides.^114^ (J) Correlation of helix dynamics with fusogenicity. Dynamics is expressed relative to L16 (=0) by the Hamming distance of the vectors of the populations of amide deuterons exchanging very fast, fast, intermediate, or slow as given in part (E). (A–E) Reproduced with permission from ref 36. Copyright 2009 Elsevier BV. (F–H) Reproduced with permission from ref 23. Copyright 2010 Elsevier BV. NOTES ON DHX MEASUREMENTS: In assessing exchange kinetics, we usually prefer the DHX mode over the HDX mode since (i) initial deuteration establishes the principal full exchangeability at amides of a given structure, (ii) protiated solvent yields a more favorable spraying behavior in ESI-MS, (iii) protiated TFE is available in purer form, and (iv) protiated solvents are more economical than deuterated ones and required in greater amounts. In a typical experiment, peptides are first exhaustively deuterated in TFE/water to achieve >95% amide exchange by incubation at 37° for days. Second, the fully deuterated peptides are incubated with a 20-fold excess of protiated solvent at pH 2.5 on ice; under these conditions, only deuterons bound to unblocked N- and C-termini and to polar amino acid side-chains are expected to back-exchange for hydrogen.^124,125^ Third, DHX is monitored at a suitable pH value (4.0–6.5) at 20 °C, depending on the flexibility of the peptide and its region to be studied. The molecular masses of the triply charged ions are subjected to various corrections to account for ionization state, the mass of charge carriers, and dilution factors as follows. The number of deuterons *D* on the exhaustively deuterated samples are determined according to *D* = 3­(*m*_D_ – *m*_H_) – 3, where *m*_D_ and *m*_H_ are the masses of the centered envelopes of the deuterated species and of nondeuterated reference samples, respectively. The number of deuterons *D* as a function of time (*D*(*t*)) in the actual DHX reaction is determined by *D*(*t*) = 3­(*m*_D_ – *m*_H_) – 0.05 × *D*_max_, where *D*_max_ is the calculated number of labile deuterons on the respective peptide (see refs 36, 127 for additional details). Is TFE a good solvent? That TFE/water mixtures stabilize the α-helical state of a peptide has been known for a long time while the underlying mechanism is still a controversial issue. It has been argued that TFE dehydrates the peptide backbone thus prohibiting the formation of destabilizing water/main-chain H-bonds, that TFE reduces the conformational entropy of hydrophobic side chains, and that TFE may indirectly stabilize a helix by destabilizing the unfolded state or by perturbing the structure of water (reviewed by refs 13, 134 ). One MD simulation of a TM-helix in a TFE/water mixture showed the frayed termini to preferentially interact with water while the hydrophobic side chains within the core of the helix interact with the apolar moiety of TFE. The TFE layer thus provides a tightly packed apolar environment facing hydrophobic side chains while allowing water molecules to assemble around polar groups.^135^ Due to its intermediate permittivity (ε = 8.55), TFE reasonably mimics bilayer polarity (ε = 4.0 to 12.4, depending on the position of a probe^74^).

### 3.3 Small Amino Acids Impact on Helix Backbone Fluctuations

As described above, assessing the impact of Gly on TM-helix geometry in 3D-structures had produced mixed results (section 2.1), while introducing multiple Gly residues clearly destabilizes host helices (section 3.1). To assess its potential promotion of conformational backbone fluctuations, Gly was placed at different positions of an LV16 host (Figure 8 A). Indeed, *overall* DHX kinetics show that a triple Gly mutant strongly accelerates DHX (Figure 8 B), whereas the impact of single-site Gly mutants was more muted.^24^ In addition, we employed a DHX protocol that was designed to assess the *site-specifc* effects of Gly.

**Figure 8:**
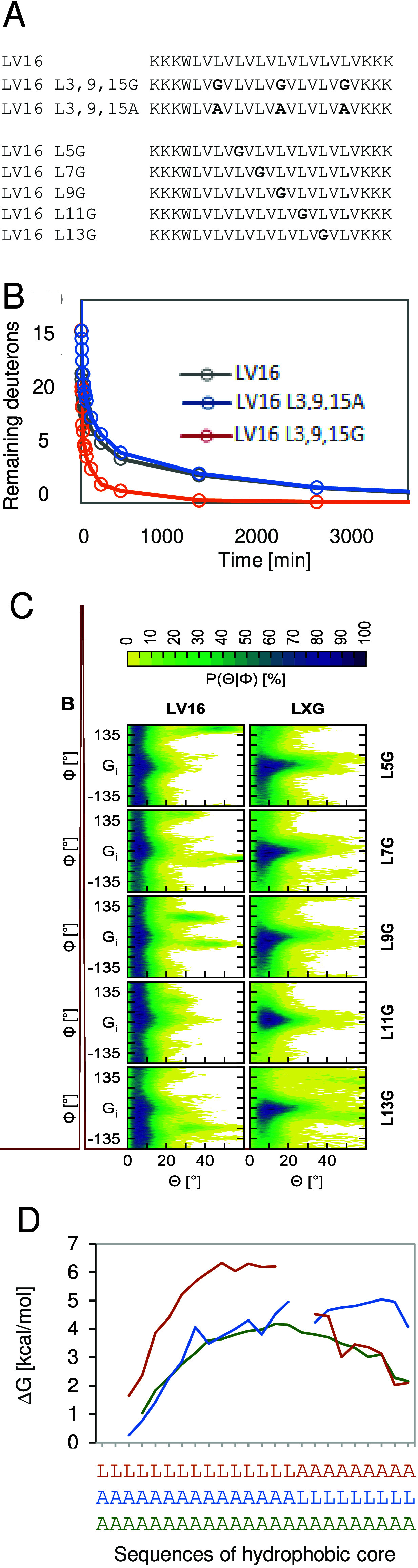
Impact of Gly and Ala on TM-helix flexibility. (A) Sequences of the LV16 derivatives used here. (B) DHX kinetics (recorded at pH 5, 20 °C in TFE/water) show that the triple Gly mutant exchanges much more rapidly than LV16 while the triple Ala mutant is indistinguishable from LV16. (C) Probability of helix bending (Θ, horizontal) or swiveling (Φ, vertical) motions as suggested by MD simulation of the single Gly mutants in TFE/water. See Figure 1A for the definition of the bending and swivel angles. (D) Flexibility profiles derived from DHX in TFE/water demonstrating high flexibility within an poly-Ala helix and within poly-Ala moieties of hybrid sequences while their poly-Leu moieties are rather rigid (Lys tripletts bordering the hydrophobic sequences are not shown, the Δ*G* values of some positions could not be determined due to some local DHX kinetics being too complex for evaluation, the flexibility profile of an L16 helix could not be calculated due to multiple overlaps of peptide fragments). (C) Reproduced with permission from ref 24. Copyright 2018 Americal Chemical Society.

To this end, peptides are fragmented in the gas phase of the mass spectrometer by electron transfer dissociation (ETD) after different periods of DHX. ETD fragmentation, done after different incubation periods, produces a series of c- and z-fragment ions that extend from a given residue position toward the N- or C-terminus, respectively (see: Figure 11). Analyzing the deuterium contents of the individual fragments reveals the deuterium distribution across a helix at different time points. These distributions uncovered gradual progression of amide exchange from the helix termini toward its center, i.e., helix fraying, as well as a differential impact of single Gly on local helix dynamics.^22^ MD simulations suggest that Gly not only introduces a packing defect but also that the latter enhances local hydration and triggers a redistribution of α-helical and 3_10_-helical H-bonds. These effects facilitate helix bending, but not swivel, motions near Gly, with consequences for the collective dynamics of the whole helix (Figure 8C). Gly residues localized to the concave face of the bends, suggesting that the removal of a side chain reduces steric hindrance to bending (Figure 1D). By contrast, the parental LV16 helix remains essentially straight during simulation (Figure 8C).^22^

By contrast to the triple Gly mutant, a triple Ala mutation did not measurably speed up overall DHX relative to LV16 (Figure 8B). We therefore also compared hybrid sequences of poly-Ala and poly-Leu. In this case, site-specific DHX rate constants *k*_exp_ were transformed into the Gibbs free energies of local unfolding after correcting them by the sequence-dependent *k*_ch_ values (which account for the modulation of DHX by effects of primary structure, see: Figure 5). This yielded “flexibility profiles” where the free energies Δ*G* characterizing the residue-specific open–close fluctuations are plotted against sequence according to the formalism given in Figure 5. The flexibility profiles reveal gradients of local unfolding energies that confirm elevated flexibility near frayed helix termini. Within helix cores, poly-Ala H-bond strengths are 3–4 kcal/mol, whereas the Leu portions exhibit strengths of up to 5–6 kcal/mol (Figure 8D). To put this into context, values of ∼1 kcal/mol had previously been reported for intrahelical H-bonds in water (remember that water can effectively compete with intrahelical H-bonding!), 2–8 kcal/mol for H-bonds of model compounds in apolar solvent (ref 136 and references cited therein), 4–5 kcal/mol for membrane-spanning protein domains,^71^ 5–6 kcal/mol for an amide H-bond in the core of a soluble protein,^137^ and 6.6 kcal/mol for H-bonds *in vacuo* (reviewed in ref 72). In addition to the energetics of pure H-bonding, a protein helix is also stabilized by side-chain interactions, as noted in section 3.1.^23,42^ Interestingly, the difference between our experimentally determined per-residue free energies of local unfolding for a poly-Leu helix (5–6 kcal/mol) vs those for a poly-Ala helix (3–4 kcal/mol, Figure 8D) equals ∼2 kcal/mol. This number is close to the sum of (i,i ± 3,4) side-chain/side-chain interaction energies calculated for the poly-Leu helix (∼1.7 kcal/mol).^23^ The numbers support the idea that the higher conformational flexibility of a poly-Ala helix relative to poly-Leu mostly results from a lack of side-chain interactions although it should be borne in mind that these values are approximations, as H-bonds are influenced by solvent polarity as well as by geometric and entropic effects.^72^ Consistent with this idea, the destabilizing effect of Ala on another type of host helix results from it elevating the local helix unfolding rate that depends on intrahelical interactions.^40^ It can thus be concluded that the free energies obtained by site-specific DHX measurements are thought to reflect the sum of H-bonding (∼3–4 kcal/mol in TFE/water) and side-chain interactions.

In sum, Gly can promote TM-helix bending motions albeit to a lesser extent than Pro. β-Branched aliphatic amino acids cause more subtle forms of helix flexibility, such as vibrational backbone motions that cause transient local amide H-bond openings detectable by DHX. The previously reported apparent near equivalent helix-promoting effects of Leu, Ile, and Val in apolar environments, as assessed by monitoring helix/coil equilibria by CD spectroscopy,^30,31^ is likely to reflect the inability of this method to detect subtle differences in vibrational backbone motions. In polar environments, the lower van der Waals interaction energies of Val relative to Leu side chains may be less important since β-branching may shift the helix/coil equilibrium by increasing side-chain entropy in the coil state.^25,38^

### 3.4 Conformationally Flexible TM-Helices Drive Lipid Mixing

Conformationally flexible TM-helices are involved in certain stages of biological membrane fusion, which is at the heart of cellular secretion and endocytosis, infection of eukaryotic host cells by enveloped viruses, cell–cell fusion etc. While most intracellular fusion events are driven by soluble NSF (*N*-ethylmaleimide-sensitive factor) attachment protein receptors (SNAREs), virus/host cell fusion depends on fusogenic viral envelope proteins. Although both types of fusogens are not related by amino acid sequence, they share basic architectural features that allow them to closely appose and finally fuse two membranes (reviewed in refs 138–143 ).

Bilayer merger is thought to result from a sequence of events that starts with membrane apposition. With intracellular fusion, soluble domains of cognate SNARE subtypes located on opposing membranes interact by way of forming a coiled coil complex that juxtaposes membranes. SNARE complex formation is regulated by a multitude of accessory factors^143^ which is not the subject of this review. In the case of viral fusion proteins, N-terminal amphipathic fusion peptides bind to the target membrane, which is followed by refolding of soluble domains, thus pulling membranes together. Close apposition requires that the repulsion between both membranes is overcome. In any type of fusion, membrane apposition is followed by lipid mixing, i.e., the rearrangement of the hydrophobic lipid tails and the hydrophilic heads in the lipid bilayer. Lipid rearrangement requires temporary disruption of the bilayer structure believed to involve high-energy intermediate(s) on the way to bilayer merger. In one intermediate, lipids are believed to splay both fatty acid tails such that one tail can be exposed at the bilayer surface, ready to contact and insert into the apposed bilayer. This results in the formation of an hourglass-shaped structure, termed the stalk intermediate. Fusion then proceeds to a hemifused state, where outer but not inner leaflets mix. Hemifusion dissolves by formation of a fusion pore whose expansion results in complete bilayer merger at a late step (Figure 9). Both, overcoming membrane repulsion as well as lipid splay are considered to require substantial energies which are lowered by fusogenic proteins that can be understood as catalysts of fusion.^143^ Their function depends both on the soluble coiled coil domains and on the single TM-helices of SNARES and viral fusogens. Replacing them by lipid anchors, mutating them, or truncating them can strongly affect bilayer mixing (reviewed in refs 141, 144, 145 ). It is thus crucial to understand how TMDs can facilitate membrane reorganization.

**Figure 9:**
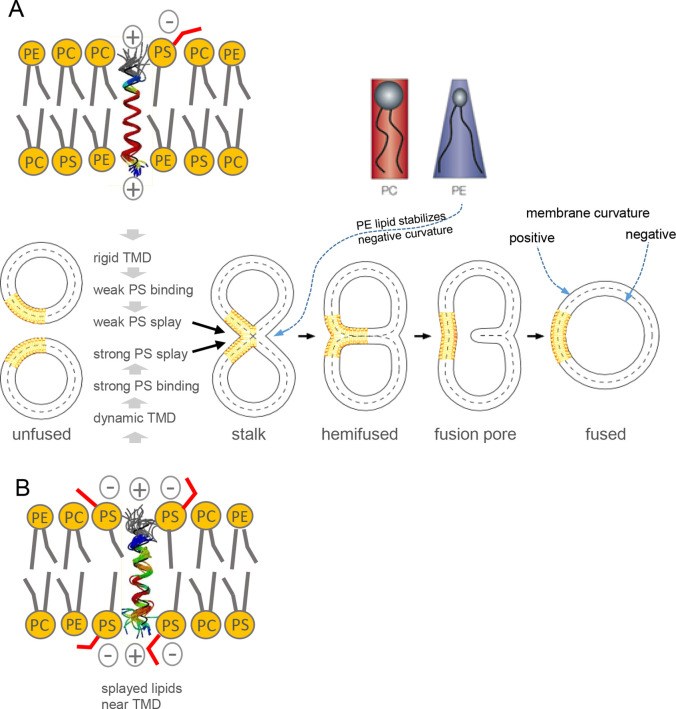
A connection between TM-helix dynamics, lipid binding, and bilayer mixing. Membrane fusion is thought to proceed through a stalk, a hemifusion intermediate, and formation of a fusion pore to complete bilayer merger (for the sake of clarity, proteins are omitted from the general model and only part of the lipids are depicted). The ability of a flexible TM-helix to bind phosphatidylserine may promote lipid splay leading to stalk formation. Enlarged bilayer patches hold either a rigid TM-helix that elicits only low levels of splay (A) or a conformationally flexible TM-helix causing strong splay (B). Encircled + and – signs denote net electrical charges of Lys side chains and PS headgroups, respectively. The local negative curvature associated with the stalk is believed to be stabilized by phosphatidylethanolamine (PE) as a result of its overall cone shape, while phosphatidylcholine (PC) is cylindrical.

Interestingly, peptidic versions of TMDs of a diverse set of fusogens display autonomous unregulated fusogenicity after reconstitution into artificial liposomal membranes. What is more, their fusogenicity is related to their conformational flexibility. For example, mutating a Gly-based motif of the Vesicular Stomatitis virus G-protein TMD decreased its fusogenicity^146,147^ as well as helix flexibility as shown by DHX.^115^ *In cellulo* experiments confirm the biological relevance of these *in vitro* experiments. Indeed, mutating the Gly motif of the full-length viral G-protein resulted in loss of syncytia formation, a marker of cell–cell fusion.^148^ In another example, synthetic TMDs of the presynaptic SNAREs synaprobrevin 2 and syntaxin 1A proved to be fusogenic *in vitro*, while the rigid Leu-based helix L16 did not.^149^ Partial exchange of SNARE TM-helix residues by Leu compromised helix flexibility^127^ as well as its fusogenicity.^149^ The fusogenicity of SNARE TMD peptides is mimicked by the LV-peptide system elaborated on in section 3.2. Indeed, fusogenicity increased with an increasing Val/Leu ratio that is connected to stronger conformational flexibility (Figure 7I,J).^36,114^ Again, the sequence-dependence of the TMD-peptide-driven liposome fusion was replicated in cellular models. Bruns and co-workers investigated hybrid versions of synaptobrevin 2 whose TMD had been replaced by low-complexity sequences. While an L16-like Leu sequence did not allow secretion from chromaffin cells, an LV16-like LeuVal repeat sequence supported the onset of fusion pore formation and its dilation similar to the original wild-type SNARE TMD. Fusion was also supported by poly-Val or poly-Ile TMDs.^133^ The conformational flexibility of these TMDs was compared by MD simulation by Böckmann and co-workers. They found the root-mean-square fluctuations of helices formed by Val or Ile to be similar to those of the synaptobrevin TM-helix and stronger than those of fusion-inactive poly-Leu. Poly-Val and poly-Ile also tended to unfold transiently and locally over the course of the simulation and did so more readily than poly-Leu.^140,150^

What could be the mechanism by which the conformational flexibility of a TM-helix contributes to bilayer merger? In the absence of coiled coil domains driving membrane apposition, TMD-driven spontaneous liposome fusion is thought to be initiated by random liposome–liposome collisions. The probability by which random liposome–liposome collision turns into fusion^151,152^ is indeed consistent with the observed ensemble kinetics of liposome fusion. Fusion in the ensemble takes many minutes^36,114,153^ while individual fusion events are complete within tens of μs.^154,155^ The probability by which membrane apposition, regardless of whether it is achieved by random collision or by soluble fusion protein domains, turns into actual lipid mixing may depend on the extent of lipid splay leading to stalk formation. Lipid splay was originally suggested by simulations^156–158^ that inspired an experiment where the abilities of two paradigmatic LV-peptides to induce liposome fusion, lipid splay, and lipid binding were investigated in parallel. Experiments were done in bilayers that were composed of dioleoylphosphatidylcholine (DOPC), dioleoylphosphatidylserine (DOPS), and dioleoylphosphatidylethanolamine (DOPE) in various combinations. Briefly, the stronger fusogenicity of the conformationally flexible LV16 relative to that of rigid L16 mainly reflects its stronger ability to induce mixing of the outer leaflet of the membrane, which leads to hemifusion. A more efficient outer leaflet mixing by LV16 relates to its stronger ability to induce lipid splay, as indicated by more frequent tail/headgroup contacts detected by NMR spectroscopy. The fusogenic function of a TMD also depends on the lipids. In detail, the differential fusogenicity of LV16 and L16 is most pronounced in the presence of DOPS; this matches the stronger binding of a DOPS derivative to LV16, relative to L16. We thus proposed that stronger DOPS binding to a TM-helix via electrostatic interactions between its basic Lys tags and the acidic PS headgroups causes stronger DOPS splay.^153^ As a result, stronger lipid splay is thought to translate into more efficient outer leaflet mixing, followed by full fusion. The importance of basic juxtamembrane domains for fusogenicity has also been demonstrated for full-length SNARES.^159,160^ That PS is crucial for highly efficient fusion is also known from other studies where PS promoted liposome fusion by SNARE TMDs flanked by their basic juxtamembrane domain^161^ or where raising PS levels enhanced an early step of SNARE-mediated fusion.^162^ Omitting DOPS, as in pure DOPC or in DOPC/DOPE liposomes, minimized the impact of TM-helix flexibility on liposome fusion. At the same time, a DOPC/DOPE mix favored peptide-independent lipid splay which enhanced unspecific fusion of peptide-free liposomes. The impact of DOPE is attributed to its cone-shape which is known to promote fusion via stabilization of the negative curvature of the stalk intermediate.^163,164^ In addition, PE may facilitate fusion by increasing lateral membrane pressure at the level of lipid tails thus promoting their splaying.^165^

The *in cellulo* analysis of hybrid synaptobrevin 2 variants revealed that conformational flexibility is only required within the N-terminal half of the TMD. The N-terminal half spans the cytoplasmic leaflet of the vesicle bilayer, which is topologically equivalent to the outer leaflet of synthetic liposomes. Thus, its conformational flexibility is well suited to cause lipid splay preceding stalk and fusion pore formation.^133^ Above that, the extent of Val and Ile over-representation in the TMDs, as revealed by a comparison of many SNARE subtypes, appears to depend on the curvature of the cellular membrane type where the respective subtype is located. That the efficiency of fusion increases with membrane curvature is well-established.^166^ Specifically, increasing fractions of β-branched residues are detected in the TMDs of SNAREs having to fuse low-curvature membranes.^133^ Mechanistically, it is thus conceivable that the lower lateral pressure exerted at the level of the fatty acid chains in a low curvature bilayer causes lower levels of basal lipid splay.^159^ It would follow that the fusion of a low-curvature membrane requires a more flexible TM-helix that is more supportive of the splay. Conversely, a more rigid TM-helix may suffice for fusion of a highly curved bilayer whose strong inherent lateral pressure causes a higher basal lipid splay. In this context, it is interesting that the low fusogenicity of the synaptobrevin/poly-Leu hybrid could be rescued by agents increasing positive curvature of the cellular membrane.^167^

Apart from the general over-representation of Val and Ile in the TMDs of SNARE proteins and viral fusogens, Gly is also overrepresented within their N-terminal half.^116,149^ Mutating a Gly, which is evolutionarily conserved within the synaptobrevin 2 TMD, alone or in combination with another small and semiconserved amino acid (Gly, Ala, or Cys) three residues downstream by Lang and co-workers strongly reduced exocytosis in cellular models. In parallel to this, the double mutation attenuated the reversible transition of the TMD from α-helix to β-sheet, used as a marker of conformational flexibility.^168,169^ MD simulations suggest that the conserved Gly introduces a flexible hinge into the synaptobrevin 2 TM-helix.^150,170^ However, hinge formation *per se* seems not mandatory for SNARE fusogenicity as the poly-Val and poly-Ile TM-helices of the strongly fusogenic synaptobrevin hybrids did not exhibit flexible hinges.^150^

Taken together, these results provide a link between TM-helix destabilization by β-branched residues and Gly and a crucial step of membrane fusion, i.e., lipid splay leading to the stalk intermediate. The available evidence suggests that the statistically over-represented Val, Ile, and Gly within the TMDs of fusogenic proteins enhance their conformational flexibility, thus leading to stronger binding of negatively charged lipid species. Possibly, the dynamics of the hydrophobic TMD cores determines the conformational space of their basic juxtamembrane domains, thus facilitating their electrostatic interaction with negatively charged lipid heads. The binding energy might offset the energy required for tail splaying, which ultimately establishes hydrophobic contact between closely apposed bilayers. As a consequence, the probability by which membrane apposition leads to bilayer merger may be crucially enhanced, also depending on the lipid composition and curvature. To our knowledge, the experimental connection between conformational dynamics, PS binding, and lipid splay is currently based on only two TM-helices, L16 and LV16.^153^ MD simulations go much further in indicating TMD-induced lipid splay around a SNARE complex.^158,171^ Future investigations are likely to close the gap between experiment and simulation and may interrogate TM-helices from SNARE proteins that differ in their contents of helix-destabilizing amino acids and hydrophobic lengths, with a view to expanding the correlation matrix. These investigations should obviously include TMDs from viral fusogens as well as their N-terminal amphipathic fusion peptides to probe the generality of the results.

### 3.5 Intramembrane Proteolysis Is Permitted by Flexible Substrate TM-Helices

In intramembrane proteolysis, peptide bonds are cleaved within the plane of a lipid bilayer by a membrane-spanning protease. This usually results in cleavage within the TMD of a substrate protein whose marked conformational flexibility supports at least two steps leading to cleavage, as outlined below. Depending on the biological context, substrate cleavage can activate membrane-tethered transcription factors and transcriptional activators, lead to the secretion of growth factors, regulate protein degradation, etc. Intramembrane proteases are distinguished by their TM-topologies and by the nature of their membrane-embedded active site. Aspartate intramembrane proteases harbor a characteristic GxGD consensus sequence within one of their TMDs, which forms the active site together with an Asp residue from a neighboring TMD. Serine intramembrane proteases, also known as rhomboids, share a Ser-His catalytic dyad. The active site of site-2-protease, a metallo-protease, coordinates zinc and that of the glutamate intramembrane protease Rce1 is based on Glu (reviewed in refs 172–174 ).

That their conformational flexibility might affect the cleavage of substrate TM-helices by intramembrane proteases was suggested more than two decades ago when proteolysis by site-2-protease was found to depend on an AspPro pair within its TMD.^175^ Also, helix-destabilizing residues, such as Gly, Ala, or Pro, were found to promote cleavage of substrates by rhomboid proteases (reviewed in ref 176). At the same time, rhomboid proteases turned out to be surprisingly slow enzymes, cleaving in the range of minutes,^177^ compared to most soluble proteases that cleave within fractions of a second. Similarly slow kinetics of intramembrane proteolysis were also demonstrated for γ-secretase.^178–180^ These findings initiated a conceptual view where the mechanism of intramembrane proteolysis, which includes formation of an enzyme/substrate complex, bond scission, and release of cleavage products (Figure 10), was hypothesized to include one or more high energy barriers that are reduced by the conformational flexibility of a substrate TM-helix.^176,181,182^

**Figure 10:**
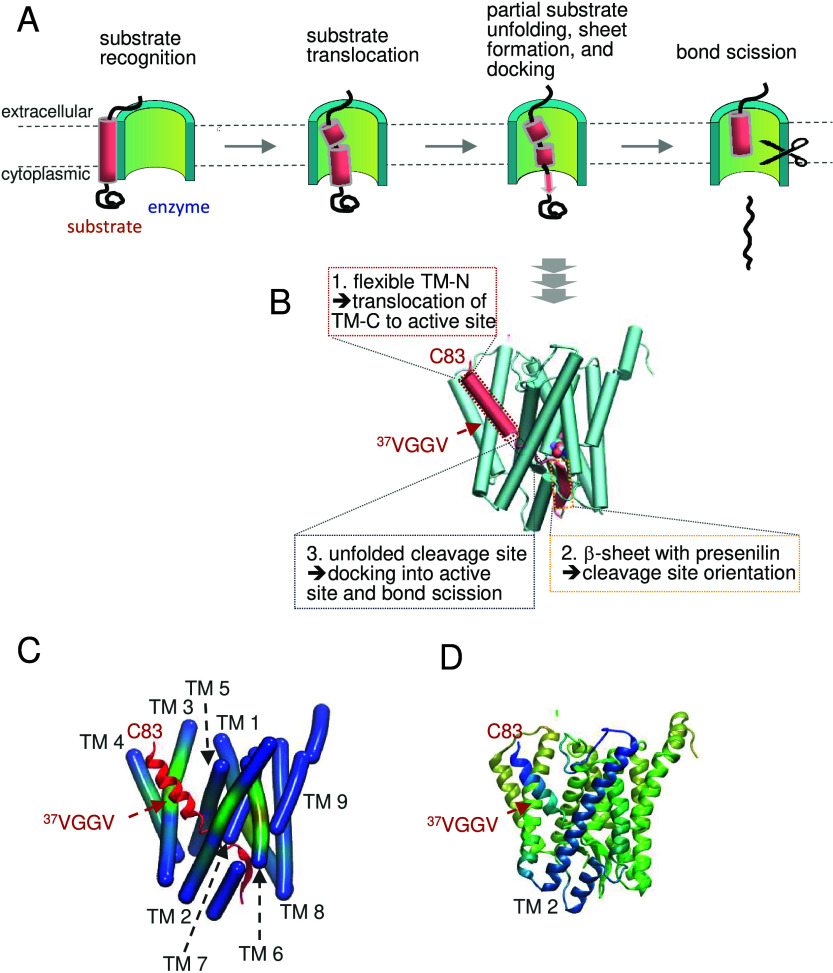
Mechanism of intramembrane proteolysis by aspartate proteases. (A) Cleavage starts with recognition of a substrate at an exosite at the protein/lipid boundary. In order to access the catalytic cleft, the substrate TMD harboring the cleavage site needs to translocate to the water-filled interior of the enzyme which is likely to be facilitated by a flexible TM-helix. Unwinding of the C-terminal part of the helix is thought to precede formation of a tripartite antiparallel β-sheet with domains of the enzyme. The sheet orients the cleavage site near the catalytic residues and promotes its docking into the cleft, thus allowing rapid bond scission. (B) The structure of the APP/C83 substrate within presenilin, the catalytic subunit of γ-secretase (pdb 6IYC^186^). Parts of the C83 TM-helix that have been shown experimentally to impact cleavage are boxed. (C) BENDIX representation of the structure, revealing significant kinks in presenilin TM-helices 1, 2, 3, and 6 (where the catalytic GXGD motif is located). Surprisingly, the kink at the VGGV motif of C83/APP shown by NMR spectroscopy in its unbound state^189,190^ is not seen in the model. However, the original electron density map of the C83 helix is also consistent with a bent helix (see figure S4F in ref 185). It should also be noted that formation of a stable complex required cross-linking of a C83 residue close to its N-terminus to presenilin. Although cross-linking preserved cleavage, it makes the orientation and the structure of the enzyme-bound substrate helix in its un-cross-linked natural form somewhat uncertain. See the caption of Figure 1 for BENDIX usage. (D) The structure colored according to the B-factors calculated from the electron density maps. *B*-Factors (increasing from green to blue) are consistent with elevated dynamics of TM2 and C83.

γ-Secretase contains the aspartate protease presenilin as its catalytic subunit. Presenilin is associated in a 1:1:1:1 stoichiometry with three other subunits, nicastrin, PEN-2, and APH-1, thus forming the γ-secretase complex. Amyloid precursor protein (APP) arguably is the most thoroughly studied γ-secretase substrate. At the first stage of substrate selection, its large extracellular domain is shedded by β-secretase or α-secretase to yield the membrane-spanning C99 or C83 fragments, respectively. Subsequent cleavage of these fragments by γ-secretase starts at ε48 and ε49 sites close to the TMD’s C-terminus and proceeds to ζ- and γ-sites further upstream, thus yielding a number of proteolytic fragments, the Aβ peptides, some of which are believed to cause neuronal degeneration associated with Alzheimer’s disease.^183,184^ Yi and co-workers solved the structures of γ-secretase complexed with C83 or C99 as well as with some of the smaller proteolytic C99 fragments by cryo-EM.^185,186^ These structures are extremely revealing, as they visualize the bound substrate helices prior to bond scission. The regions encompassing the respective cleavage sites are unfolded and followed by three to five residues that form a tripartite β-sheet with two short segments of presenilin.

One crucial question in the field of intramembrane proteolysis is how an intramembrane protease distinguishes its substrates from an abundance of nonsubstrates that are simultaneously present in a membrane. Apart from APP C83 and C99, around 150 other γ-secretase substrates have been identified (reviewed by ref 187). All of these are single-span proteins with a *N*_out_ topology and a small ectodomain, usually generated by initial shedding. However, no clearly recognizable consensus sequence is shared by the TMDs of these substrates that could guide their recognition by the enzyme. One speculation was that substrate selection from the ∼10-fold larger pool of single-span *N*_out_ proteins in the human genome might depend on enhanced helix flexibility around the cleavage sites, downstream of which helix-destabilizing amino acids are often found.^188^ A related question asked whether the remarkably slow turnover of substrates, as noted above, might be explained by the mechanism of their selection.^178–180^

In order to gauge which role substrate TM-helix dynamics might play in answering these pertinent questions, the results of cleavage assays were connected to the outcomes of DHX, NMR spectroscopy, and MD simulation of the model substrates. Figure 11 illustrates how the flexibility profile of APP TM-helix was obtained by DHX-MS. DHX in a TFE/water solution (Figure 11A) produced mass spectra diagnostic of mainly uncorrelated exchange in the EX2 mode (Figure 11B). Gas phase fragmentation led to fragments (Figure 11C) from which DHX kinetics at the resolution of single residues were calculated (Figure 11D). Amides at frayed helix termini and at residues G38 and V39 within a flexible hinge (see below) tended to give rise to biphasic DHX kinetics whose slow phase was used to compute the *k*_exp_ distribution (Figure 11E, see the caption for details). This distribution was transformed to the flexibility profile (Figure 11F).

**Figure 11:**
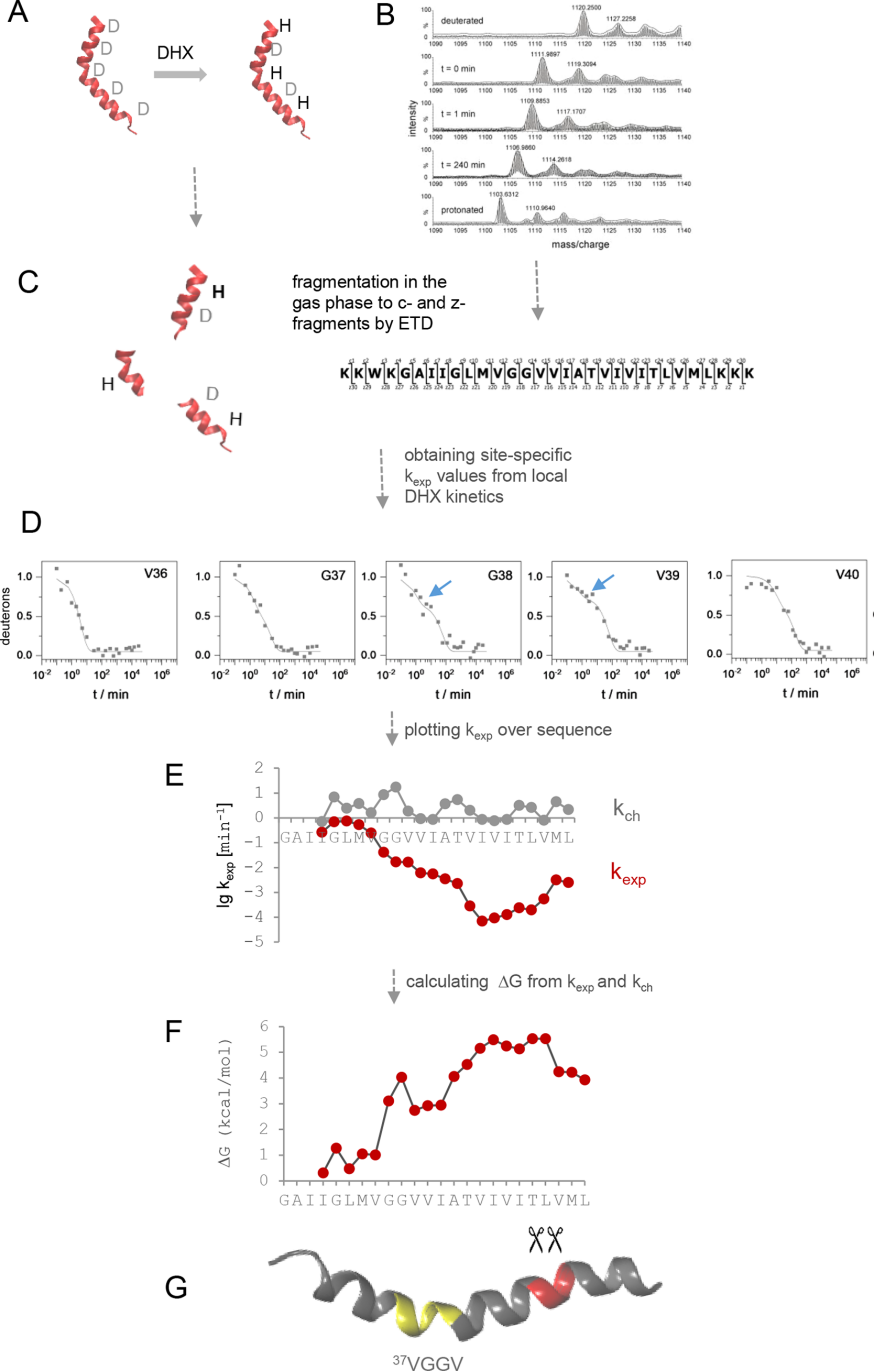
Determining site-specific free energies of backbone fluctuations of the APP TM-helix by DHX. (A) DHX of a kinked helix in TFE/water starts at frayed termini and at the flexible hinge. (B) Isotopic envelopes that gradually shift with ongoing DHX signify preferential uncorrelated exchange in the EX2 regime. (C) Fragmentation of peptide ions in the gas phase by ETD after different time points of DHX. Cleavage is induced by an electron donor (1,4- dicyanobenzene) between nitrogen and Cα carbons and produces a series of c- and z- fragments. (D) Subtracting the masses of consecutive fragments from each other yield the deuterium contents at individual amide nitrogens as a function of time. Only some exemplary cases are shown. The hinge residues G38 and V39 exhibit biphasic exchange kinetics whose fast phase (arrows) may result from a superposition of dominant uncorrelated exchange (EX2) with rare correlated exchange reactions (EX1) in a mixed EX1/EX2 mode, as detailed in the supporting information of ref 192. By contrast, the slow phase is thought to exclusively reflect uncorrelated EX2 exchange. (E) Distribution of *k*_exp_ and *k*_ch_ over primary structure (only the hydrophobic part of the triple-Lys-tagged peptide sequence is covered). The differences between both rate constants reflect the extent of protection from amide exchange by H-bonding at a given site. (F) Distribution of Gibbs free energies of local helix unfolding, the flexibility profile. Values are taken from ref 192. (G) NMR structure of the APP TMD (pdb 6YHF^190^) aligned with the flexibility profile in part (F). Scissors denote the ε48 and ε49 cleavage sites.

Interestingly, the Δ*G* values form a trough at the VVI sequence downstream of a VGGV motif within the N-terminal half (TM-N) of the APP TMD. The trough indicates weaker H-bonding resulting from lower strengths of packing interactions across the GlyGly pair to upstream residues.^191,192^ Indeed, the VGGV motif marks a permanent kink detected by NMR spectroscopy by Sanders and co-workers in a micelle^189^ and in the isotropic environment of TFE/water by Muhle-Goll and co-workers^190^ (Figure 11G). MD simulations suggest the kink forms a flexible hinge where the helix performs bending and twisting motions. These motions are facilitated by locally interconverting α-helical and 3_10_-helical H-bonding.^193,194^ Importantly, mutating kink residues changes flexibility and results in strongly reduced cleavability by γ-secretase.^191^ At a similar location, γ-secretase substrate Notch 1 holds an AAAA stretch. Replacing AAAA by LLLL strongly stabilizes the helix and results in reduced cleavage while replacing AAAA by GGGG destabilized the helix and facilitates cleavage (Figure 12A,B).^195^ The AAAA motif does not give rise to a kink, however. Rather, NMR spectroscopy revealed a continuously bent Notch 1 TM-helix in TFE/water. Fitting the restraints on short residue–residue distances and torsion angles, as obtained by NMR, were used to generate bundles of helices that reflect the potential conformational diversity of a given helix. Accordingly, the AAAA→LLLL mutation reduced the conformational spread, while the AAAA→GGGG mutant is disordered and its TMD is subdivided into two short independent helices (Figure 12C). Consistent with the differential conformational flexibilities around the AAAA, LLLL, or GGGG motifs, DHX detected slight, no, or substantial local biphasic exchange kinetics (see ref 192, figure S1 for additional examples of biphasic DHX). Biphasic amide exchange kinetics therefore qualify as a marker of locally unfolded states.

**Figure 12:**
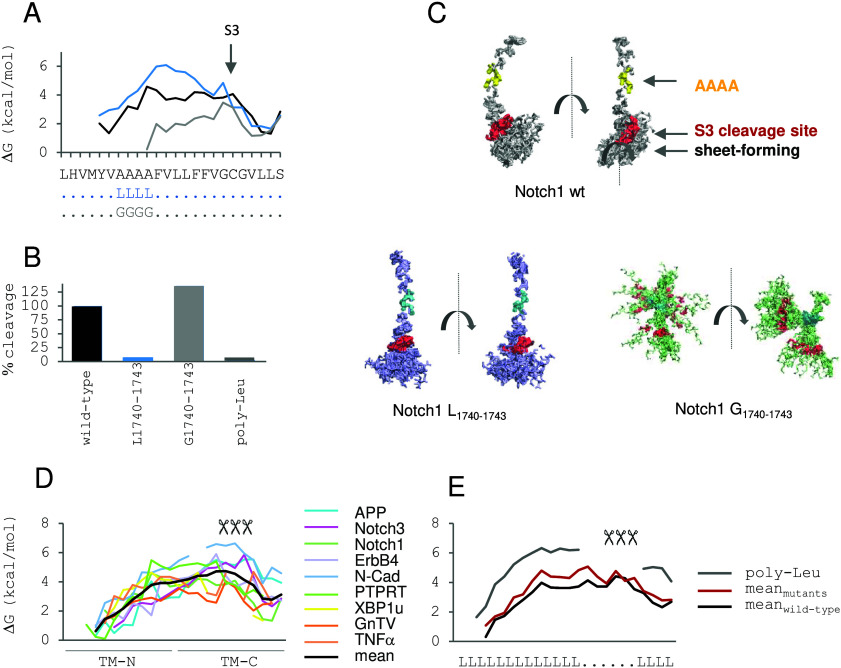
Relating the cleavability of substrate TM-helices to their conformational flexibility. (A) The flexibility profile of the Notch 1 TM-helix recorded in TFE/water changes drastically upon mutating its AAAA motif to LLLL or GGGG. (B) Cleavability by γ-secretase is reduced by the helix-stabilizing AAAA→LLLL mutation to the level of the nonsubstrate poly-Leu TMD, but enhanced by the destabilizing AAAA→GGGG mutation. (C) Structural bundles obtained by NMR spectroscopy reveal substantial conformational variability of the wild-type Notch 1 TM-helix (pdb 8or5). The AAAA→LLLL mutant (8ory) exhibits less variability while the AAAA→GGGG mutant (8orz) is much more disordered than the wild-type. The structures were superimposed onto TM-N to provide an estimate of the conformational spread of TM-C in an enzyme/substrate complex where TM-N is bound to enzyme TMDs. Data from.^195^ (D) For an overview, the flexibility profiles of the hydrophobic sequences of nine aspartate protease substrate TMD peptides are overlaid and averaged (in black). Note the trend to higher flexibility within TM-N relative to TM-C (γ-secretase substrates: APP,^192,208^ Notch 1, Notch 3, ErbB4, and *N*-cadherin,^192,195^ SPP substrate: XBP1u,^201,238^ SPPL2a substrate: TNFα,^203^ SPPL3 substrate: GnTV^204^). (E) For those substrates investigated by point mutagenesis, the mean flexibility profiles of wild-type and those mutant sequences that exhibit >50% reduced cleavability by the respective protease are compared (APP plus G38L and G38P mutants,^208^ Notch 1 plus AAAA→LLLL mutant,^195^ XBP1u plus mt1, mt2, and mt5 ^201,238^ , GnTV plus G22/26L mutant,^204^ TNFα plus AGA/LLL mutant^203^). The flexibility profiles of poly-Leu parts of Leu/Ala hybrid sequences are taken from Figure 8D to provide a benchmark for a rigid and cleavage-resistant sequence.^192,195^ Only the unfolding energies of those Leu residues given underneath the graphics are given. The scissor symbol denotes the approximate position of initial cleavage sites if known (APP, Notch 1, Notch 3, GnTV, TNFα, ErbB4). Note the tendency of cleavage-impairing mutations to increase the rigidity of substrate helices. NOTES ON DHX-MS: TFE/water was chosen as a reasonable mimic of the interior of an intramembrane protease. Due to the similar polarities of TFE (ε = 8.55) and protein interiors (ε ∼ 4 to 12 corresponding to dry and internally solvated proteins, respectively^196^), TFE is considered a reasonable mimic of the water-filled catalytic cleft of γ-secretase^197,198^ and likely of other aspartate proteases. The TFE/water mixture also maintains helicity and prevents aggregation of the TMD peptides. During DHX, the previously used collision-induced gas phase fragmentation of peptides leads to hydrogen scrambling where hydrogens transfer during fragment formation, thus prohibiting the precise determination of the site-specific extent of DHX or HDX.^199^ The more recent ETD fragmentation minimizes this problem.^200^ Gas phase fragmentation techniques had originally been developed to sequence short peptides (typically around 10 residues) for the sake of identifying proteins. When larger peptides (∼30 residues) are fragmented at different time points of DHX, as done in our work, the resulting fragment spectra are exceedingly complex. These spectra contain dozens to hundreds of different c- and z-fragments, distinguished by the number of residues, charge state, and deuteration state. To facilitate their evaluation, we developed the ETD fragment analyzer. Theory and detailed evaluation procedure are described in the supporting information of ref 201 and in the manual that accompanies the code which is available at DOI 10.5281/zenodo.7223537. Briefly, the semiautomated evaluation consists of the following steps: (i) The c- and z-fragment ions to be included are determined. (ii) After DHX and ETD, the expected isotopic patterns of the fragment ions are predicted. (iii) The predicted isotopic patterns are compared to the experimental ones to determine the respective extents of amide deuteration. (iv) To increase precision, a smoothing and interpolation procedure is applied. (v) The numbers of amide deuterons as a function of exchange period *D*(*t*) is used to compute the residue-specific first order exchange rate constant *k*_exp_ *D*(*t*) = 0.95*e exp­(−*k*_exp_*t) + 0.05 (considering 5% remaining deuterated solvent after 20-fold dilution used to induce DHX). (vi) The site-specific free energies Δ*G* of local unfolding are finally computed from the equilibrium constants *K*_exp_ based on the Linderstrom–Lang theory, as detailed in Figure 5. It should be noted that the values obtained with this procedure are an upper estimate of the true values since (i) the molarity of water in 80% (v/v) TFE solvent is only 20% of the bulk molarity used for the determination of the reference chemical exchange rates *k*_ch_ and (ii) the hydration of residues in the hydrophobic core of a TMD is possibly reduced relative to the bulk phase. Both factors likely reduce *k*_ch_. In addition, TFE might influence the autoionization constant of water and *k*_ch_.^63^ In order to obtain full DHX kinetics of highly flexible or rigid parts of a helix, incubation periods from seconds to weeks can be mimicked by lowering the pH to 4.0 or elevating the pH, respectively, and correspondingly correcting the actual incubation periods.

Investigating the impact of primary structure on flexibility and cleavability was extended to the substrate TM-helices from other aspartate proteases, namely, signal peptide peptidase (SPP) and SPP-like enzymes. SPP/SPP-like intramembrane proteases have an inverted TM-topology and regulate a wide spectrum of intracellular processes.^202^ Examples include the substrate XBP1u where stabilizing its TM-helix by multiple Leu mutations virtually abolished its cleavability by SPP.^201^ The TM-helix of the SPPL2a substrate TNFα contains an AGA motif, which introduces a slight bend and signs of 3_10_-helicity. Mutating AGA to LLL stabilized the helix and strongly decreased cleavage at a downstream site.^203^ In yet another case, the TM-helix of GnTV, an SPPL3 substrate, is less ordered and rather unstable between the Gly residues of a central GxxxG motif. This part becomes more helical by mutating Gly to Leu, also resulting in reduced cleavability.^204^

Averaging the DHX-derived flexibility profiles of these nonhomologous substrate TM-helices reveals that their N-terminal halfs tend to exhibit higher mean flexibility than their C-terminal halfs (Figure 12D). Mutations that compromise cleavability at sites located close to the C-termini increase mean Δ*G* by up to ∼1 kcal/mol and do so mainly at positions several residues upstream of the initial cleavage sites (Figure 12E). As detailed above, these mutations mostly correspond to replacements of helix-destabilizing residues (Gly and Ala) by the helix-stabilizing Leu. Thus, cleavability tends to be associated with an elevated helix flexibility upstream of a cleavage site.

We note that an experimental approach that is methodologically different from the DHX protocol used in our lab had been proposed where site-specific isotope fractionation factors Φ are obtained from the ratio of NMR-derived HDX and DHX amide exchange rate constants. This ratio corresponds to the stability constant that reflects the state at equilibrium. Generally, the Φ value reports the isotopic preference in a given H-bond and it is assumed that the enrichment of deuterium at an amide, i.e., a high fractionation factor, at equilibrium indicates weak H-bonding.^205^ Although the profile of exchange rate constants initially obtained for the APP TMD by Bowie and co-workers^206^ qualitatively matches the one shown in Figure 11E,^191^ the subsequent calculation of Δ*G* values using the Φ-value approach suggested the TM-N helix to be more rigid than parts of the C-terminal half (TM-C) of the helix.^206^ A careful re-examination of the theoretical underpinnings suggests, however, that Φ-values derived from the *k*_exp,HDX_/*k*_exp,DHX_ ratios by Cao et al. reflect the stability of an amide-to-solvent H-bond, rather than the stability of an intrahelical amide-to-carbonyl H-bond for a number of reasons detailed in ref 64. This formalism thus appears to lead to erroneous results, possibly also obscuring other results where this approach reported surprisingly weak H-bonding.^75^ That solvent-exposed amide hydrogens can have surprisingly low fractionation factors had previously been noted.^205^

What is the mechanism by which the conformational flexibility of a substrate TM-helix promotes cleavage? For one thing, substrate TM-helix flexibility might facilitate its entry into the enzyme and access of its catalytic cleft.^192,203,207,208^ To substantiate this idea, the NMR-based structural bundles of wild-type and mutant APP or Notch TMDs (Figure 12C) were modeled into the known structures^209,210^ of the respective γ-secretase/substrate complexes.^190,195,211^ Interestingly, the cleavabilities of the substrate variants roughly matched the average proximities of their TM-C residues that are to form the tripartite β-sheet with presenilin to their eventual locations in that sheet. In other words, the VGGV or AAAA motif appears to be crucial in presenting the sheet-forming residues of the substrate to the cognate presenilin domains. Therefore, a flexible TM-N helix may foster initial cleavage by facilitating its translocation from the enzyme/membrane boundary to a cleavage-competent location. These results also reveal that the more detailed spatial information provided by NMR provides an even more precise link between helix dynamics and cleavability than the flexibility profiles obtained by DHX analysis.^195,211^

In line with a requirement of TM-helix flexibility, a rigid poly-Leu TM-helix (see section 3.2) expressed within the framework of soluble APP or Notch domains proved to resist cleavage. In a gain-of-function approach, grafting Gly-based motifs onto poly-Leu TM-N partially restored cleavability. Full recovery of cleavage, however, required the additional grafting of the initial cleavage sites and the sheet-forming regions of the TM-C.^192,195^ Apparently, these three TM-regions cooperate in conferring cleavability.

Does the cleavage-promoting effect of TM-C also depend on helix flexibility? Although the cleavage site has to unfold for bond scission to occur, it is still open whether or not the primary structures around them have evolved for low helix stability, as is believed for substrates of soluble proteases.^212^ For example, mutations at the APP ε-sites or at the Notch 1 S3 cleavage site to Pro or Gly do not universally promote cleavage of their TMDs; similarly, the presumed helix-stabilizing or destabilizing nature of other mutations near the scissile bonds in other substrates fail to demonstrate a clear link between local helix flexibility and cleavability.^192,195,203,213–219^ Thus, the rate of helix unfolding around cleavage sites has apparently not been optimized during evolution, indicating that it does not constitute a rate-limiting step in intramembrane proteolysis. Indeed, helices in aqueous solution unfold at nanosecond time scales,^220–222^ which is orders of magnitude faster than notoriously slow intramembrane proteolysis^177,179,180,223^ and thus unlikely to limit its rate. Rather, the mutation-sensitivity of cleavage sites might reflect the kinetics of tightly docking the unfolded substrate into the catalytic cleft of presenilin^224,225^ prior to bond scission.

Yet another mutation-sensitive region is the sheet-forming part near the TMD C-terminus where introducing the sheet-stabilizing Val^226^ facilitates Notch and APP cleavage while mutating to Leu abolishes APP cleavage.^195^ Although the formation of a β-sheet from covalently connected strands may take only tens of microseconds,^227^ the assembly of sheet from disconnected strands may take minutes.^228^ Enhancing the rate of formation and/or the stability of the tripartite β-sheet^216^ may indirectly favor cleavage at unfolded cleavage sites by orienting them between both catalytic Asp residues.

Finally, mutational studies were complemented by comprehensive sequence statistics. A systematic comparison of well established substrates to a reference group of presumed nonsubstrates has revealed several physicochemical features of amino acids that mark substrate TMDs. Crucial amino acid features mainly accumulate at the C-terminal sheet-forming positions of substrate TMDs where residues predominate that are prevalent within α-helices as well as β-strands. These include Leu, Met, Phe, Ser, and Trp.^112^ When present in the right combinations with other residue types, these amino acids may support the helicity of unbound substrate TMDs while allowing for sheet formation once they are engulfed by the enzyme. Albeit the accumulation of features is less pronounced within the TM-N of substrates, residues with short side chains, as in the VGGV or AAAA motifs discussed above, are prevalent and able to facilitate substrate engulfing by TM-N helix flexibility. Features, such as side-chain length and accessibility, mark the C-terminal juxtamembrane regions of substrates.^229^ The mutational sensitivity of the corresponding basic residue motifs within the juxtamembrane region of substrates is well documented. Basic residues may support cleavability by firmly anchoring a substrate within the enzyme.^224,230–232^ Based on these overrepresented features, machine learning predicted the entire human γ-secretase substrate scope and most of the newly predicted candidates were experimentally validated.^229^

Altogether, different parts of the TM-helix may cooperate in permitting cleavage of a single-span protein by an Asp protease. Good cleavability, such as in the case of APP or Notch 1, appears to result from (i) a flexible TM-N that facilitates the transfer of the cleavage site(s) from an initial contact site at the enzyme/lipid boundary beyond sterically obstructing TMDs of an enzyme to its interior and assist presentation of its sheet-forming C-terminal residues to the cognate enzyme domains, (ii) a TM-C that is able to switch from a helix to a sheet that stabilizes the locally unfolded state and orients the cleavage site between the catalytic residues, and (iii) efficient docking of a cleavage site into the catalytic cleft. The relative weight of these factors may help to define the position of a given candidate within a wide rank order of cleavage kinetics, ranging from good substrates to virtual nonsubstrates. To date, the cleavability of the vast majority of known substrates has been investigated by end point analysis. The much more laborious Michael–Menten cleavage kinetics have only been determined in a few cases.^178–180,233^ Future exploration might thus aim at a comprehensive comparison of the catalytic turnover numbers of a larger number of substrates with the flexibility profiles and NMR structures of their TM-helices in order to obtain a more complete mechanistic picture.

Only little is currently known about the conformational transitions on the part of the enzyme during substrate processing. In the γ-secretase/substrate complexes, most presenilin TMDs are kinked or curved, suggesting their structural flexibility (Figure 10 C). To date, no definitive substrate-free apo-structure of γ-secretase is known. When determined in the absence of a covalently attached substrate, a consensus structure of γ-secretase has been decomposed into different structures, one of which appears to contain substrate. These substructures differ mainly in the orientation of TMDs 2 and 6 of presenilin.^234^ Indeed, in the enzyme/substrate complexes, TMD 2 and the substrate TMD have the highest B-factors of all TMDs (Figure 10 D).^209,210^ This is consistent with elevated flexibility of TMD 2 and the substrate although the physical meaning of B-factors in cryo EM structures is less clear-cut than in X-ray structures.^235^ TMD 2 is believed to be crucial in substrate recognition and translocation (refs 236, 237 , and references cited therein). The ways by which the presenilin TMDs rearrange during the functional cycle of γ-secretase are still an open question.

## 4 General Conclusions and Future Perspectives

As we have seen, the conformational flexibility of individual TM-helices can be a complex mixture of side-chain rotations, vibrational backbone motions, helix bending and twisting, which is frequently associated with locally interconverting α-, 3_10_-, and π-helical states, as well as of disruptions resulting in local coil regions. These motions strongly depend on primary structure, specifically on the volume of a side chain, the chemistry of its branching, its potential to H-bond with the main chain, and of course its cyclic nature in the case of Pro. Occurring within a time frame of picoseconds to nanoseconds they modulate the free energy of a given structure. In the case of an active state, these types of limited conformational flexibility are frequently associated with function, such as channel gating or substrate proteolysis.

A membrane protein built of several TM-helices, such as in a multipass protein and/or oligomer, frequently exists in different structural substates that are distinguished by biological functionality. Interconversion of substates frequently involves a redefinition of TM-helix–helix packing. In general, different states tend to be in equilibrium which each other even without an activating biological trigger as exemplified by ligand-gated ion channel proteins, where agonist-independent occasional pore openings can be observed.^106^ The relative populations or lifetimes of the different substates in a functional cycle inversely correspond to the free energies of their respective structures (Figure 13A).^8,239^ Free energy is the aggregate of enthalphic and entropic contributions to the whole system of a protein, complete with its environment. These contributions include intra- and intermolecular forces and motions. They also include biological triggers that favor an active state such as substrate or ligand binding or chromophore isomerization.

**Figure 13:**
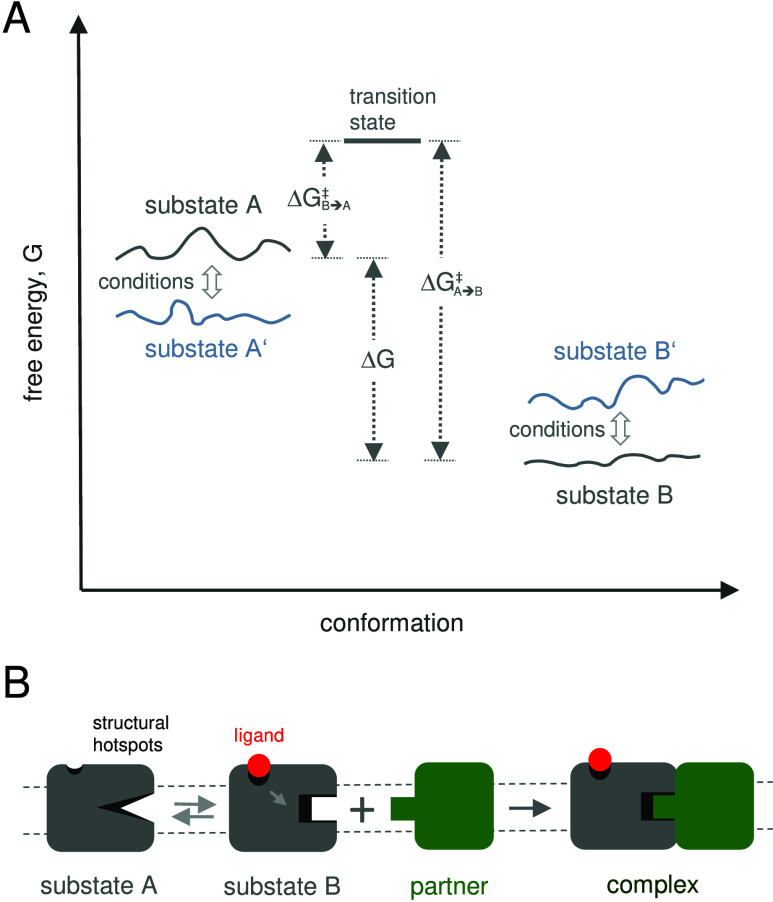
Protein substates. (A) Simplified energy diagram of a hypothetical protein with folded substates A and B that are represented by cross sections of the protein’s energy landscape at both energy minima. The lower mean free energy of substate B may result from binding of a ligand or a substrate. The mean free energy of a given substate may be shifted from A to A′ and from B to B′ or *vice versa* by environmental conditions, such as changing bilayer thickness or curvature, lipid binding etc. Mean free energies determine the populations of the substates, provided that the energetic barrier to the transition state (Δ*G* ^⧧^, shown only for substates A and B) is overcome by thermal energy. Values of Δ*G* ^⧧^ define the velocities of the reaction (usually μs to ms) from substate A/A′ to B/B′ or *vice versa*. Moreover, the free energy of a given substate is modulated by fast (ps to ns) local conformational changes, such as side-chain rotations, backbone vibration, or helix bending motions. In the active substate, these modulations may facilitate proton movement, channel opening, substrate cleavage, etc. (B) Hypothetical model of how conformational dynamics might influence membrane protein/protein interaction. In the model, the allosteric coupling between two structural hotspots at, e.g. a ligand-binding site and a protein recognition site, respectively, may underly the interconversion between substates A and B. Thus, stabilization of substate B by a ligand might promote complex formation.

The kinetics by which different substates interconvert within a functional cycle typically ranges from microseconds to milliseconds. They are determined by the activation energies separating different substates from each other via energy-rich transition states. On the part of a protein, activation energies contain the energetic costs of altering intra- and/or interhelical packing interactions and of corresponding transient H-bond openings. To minimize activation energy, rapidly functioning membrane proteins are thus often loosely packed^86^ and/or locally unfolded.^52^ Loose packing and local unfolding compromises stability, however, thus increasing the probability of denaturation.^240^

In order to better understand of how packing and its temporal changes affect function, one might systematically study the connection between the recognition of a biological trigger, the communication of this event to a functional site, and activation of function. One approach addressing these issues might focus on the regions around the readily identifiable noncanonical elements of TM-helices noted in section 2.1. In a first step, one might estimate the functional importance of these regions by comparing the accumulation of benign nonsynonymous single nucleotide polymorphisms (suggest low functional relevance) relative to the accumulation of pathogenic or gain-of-function mutations (suggest high functional relevance). In a second step, one may establish to what extent various parameters of packing are correlated to each other at these functionally relevant regions. These parameters may include α-, 3_10_- and π-helical secondary structure, physicochemical properties of local amino acids, aberrant local H-bonding patterns, intra- and interhelical TM helix–helix packing (packing density, helix–helix crossing angles), etc. In a third step, one might assess whether structural hotspots, thereby defined, are organized in distinct patterns within membrane proteins, how these potential patterns depend on their structural class, and how they differ in the respective substates.

### 4.1 Membrane Protein Dynamics and the Lipid Bilayer

The stability of a given substate may be fine-tuned by transient lipid binding to cavities and/or unsatisfied H-bond partners. The impact of lipids is an aspect that has not been touched on so far in this review. Despite a wealth of data having been accumulated during the past two decades, this area nonetheless offers much promise for future investigation (reviewed in refs 164, 241–244 ). In one example, the GlpG rhomboid protease, cavities within loosely packed domains indeed appear to facilitate the gating movements of TMDs and loops. Interestingly, destabilization by cavity formation appears to be compensated for by weak lipid binding which can thus fine-tune the balance between stability and flexibility for optimal activity.^245^ One may speculate that lipid binding has an even stronger stabilizing impact on a partially unfolded transition state. If so, then lipid binding might reduce energy barriers between substates, thus accelerating large-scale conformational changes within a functional cycle. In other words, bound lipids may function as catalysts that smooth the energy landscape of a protein. A similar function may be ascribed to internal water molecules stabilizing non-α-helical elements of TM-helices by H-bonding to polar main-chain atoms^94,246^ or cavities, similar to soluble proteins.^247^ However, water is scarce within the hydrophobic core of a lipid bilayer and therefore rare as an alternative H-bond partner for a flexible TM-helix backbone. The flexibility of a TM-helix will thus be most pronounced in polar microenvironments, such as within a hydrated intramembrane protease, as part of a fusion pore, as constituent of an ion-conducting pore, or as part of an exposed ligand-binding site formed by TMDs.

Changes of the free energy of a substate that exceed those effected by lipid binding are often achieved by changes in bilayer structure in a number of ways. “Hydrophobic mismatch” is one way by which a membrane can affect an embedded protein. In this situation, the thickness of a bilayer, as determined by the length and the degree of saturation of phospholipid acyl chains as well as by cholesterol content, is at odds with the length of a protein’s hydrophobic TM-helices. To avoid an energetically unfavorable “positive mismatch” (TM-helix too long) situation, a membrane protein may respond to bilayer thinning by stronger tilting of TM-helices relative to each other, thus altering the geometry of helix–helix packing. Also, the stability of a given substate may respond to changing membrane lateral pressure.^164^ For example, a drastically reduced lateral membrane pressure, as induced by an increased osmotic pressure in a bacterial cell, favors relaxation of the closed MscL water channel to a hugely different conformation with a widely open pore.^102,104,105^

The lateral pressure of a bilayer is described by a profile that is defined by the volume assumed by the lipid headgroups relative to the volume of their tails. Small headgroups, as in DOPE lipid, increase pressure at the level of the tails, which can stabilize an hourglass-shaped protein substate with a reduced diameter close to the middle of the membrane. The lateral pressure profile also responds to membrane curvature that changes with the diameter of subcellular vesicles upon their regulated fusion. The ability of a TM-helix to induce fusion via lipid splay, as proposed for SNARE proteins,^133,153,248^ depends not only on its own conformational flexibility but also on the shape of the surrounding lipids, in yet another example demonstrating functional interplay between protein dynamics and bilayer structure. Apart from supporting lipid bilayer mixing, SNARE TMDs may contribute to other steps along the membrane fusion trajectory.^141,249^ These contributions may include facilitation of membrane apposition through membrane thinning^250^ and promotion of fusion pore formation.^251,252^ Even after a long debate, it is still unclear whether fusion pores are primarily lipidic or proteinaceous in nature.^143^

Given the impact of lipids on membrane protein function, it is not surprising that many structures contain phospholipids and/or cholesterol bound to the periphery of a TM-helical bundle or in between the TM-helices.^253–255^ It should be borne in mind, however, that determining the vast majority of membrane protein structures known to date involved solubilization of the protein in detergent prior to crystallization for X-ray analysis or reconstitution into artificial bilayer systems for cryo-EM, such as lipid nanodiscs. It is therefore unclear to what extent these lipids represent native lipids at their original binding sites. Only few examples are known where embedding in the native membrane was preserved during cryo-EM, including the nAChR^107,110^ and bR.^94^ In addition, McKinnon and co-workers recently found that the structure of the Slo1 ion channel protein, as determined in vesicles derived from the plasma membrane of overexpressing HEK cells without prior solubilization, exhibits a better ordered, more stable, and slightly expanded TM-helix bundle compared to the structure determined after detergent treatment. Without detergent, a myriad of cholesterol and phospholipid molecules were bound to the periphery of the TM region as well as between some TMDs. The authors speculate that the association with native lipids preserves a more natural state of the protein.^256^ In the future, HEK cells might be engineered to express native lipids relevant to given membrane proteins. Structure determination in the presence of native lipids may be crucial to better understand the interplay between lipid binding and membrane protein dynamics.

### 4.2 Membrane Protein Dynamics, Packing, and Recognition

Within multipass or oligomeric proteins, the dynamics of an individual TM-helix is influenced by packing to its neighbors which depends on its primary structure.^131^ For example, Gly and other small amino acids (Ala, Ser, Thr) are preferred at TM-helix–helix interfaces where they tend to decrease the distance between the helix axes,^257–260^ consistent with the overrepresentation of small amino acids in well-packed regions.^84^ As strengthening of helix–helix packing is likely to rigidify a protein, Gly might thus decrease the flexibility of well-packed helices, although it promotes the flexibility of a free TM-helix. As noted above, stronger packing is likely to slow substate interconversions that redefine TM-helix–helix interactions. Apart from effects such a slow down might have on the function of a monomer, slower substate interconversion might indirectly reduce the kinetics of membrane protein–protein interactions. Most membrane proteins exist as dimers or multimers. The mutual recognition between membrane proteins can rest on complementary surfaces of individual TM-helices and/or on the overall spatial complementarity of TM-helix bundles.^261,262^ That the molecular shapes of pre-existing substates vary and thus define their binding competence has indeed been demonstrated for a number of soluble proteins^263,264^ and some peripheral proteins interacting with integral membrane proteins.^265,266^ In line with these isolated findings, it has been proposed that proteins at crucial nodes of functional networks display a superior degree of conformational change that may facilitate their interactions with multiple partners.^267^

Although evidence is still scarce for a corresponding conformational-selection mode of interaction within the plane of a membrane, hypothetical scenarios can be conceived where only certain substates enter complexes with themselves or partners via binding-competent arrangements of TM-helices (Figure 13B). Carrying these speculations a little further, one may wonder how proteome-wide modeling of membrane protein complexes could benefit from the comprehensive prediction of substate structures. The neural-network-based “AlphaFold” algorithm has provided a full spectrum of reliable protein models.^268^ Further, modeling individual structures has recently been expanded into predicting their interactions with a variety of binding partners.^269^ However, these algorithms still fail to accurately reflect the repertoire of substates,^270^ thus limiting their ability to faithfully predict the full set of their potential protein–protein interactions. Promising attempts to obtain reliable substate structures from AlphaFold have been presented,^270,271^ however. In addition, advanced simulation techniques have been developed to overcome the energy barriers between different substates.^272^ Amen.

## Abbreviations

APP: amyloid precursor protein
bR: bacterorhodopsin
CD: circular dichroismcircu
cryo-EM: cryogenic electron microscopy
DOPC: dioleoylphosphatidylcholine
DOPS: dioleoylphosphatidylserine
DOPE: dioleoylphosphatidylethanolamine
DHX: deuterium/hydrogen exchange
ESI-MS: electrospray ionization mass spectrometry
ETD: electron transfer dissociation
MscL: mechanosensitive channel of large conductance
nAChR: nicotinic acetylcholine receptor
MD: molecular dynamics
pdb: Protein Data Bank
SNARE: soluble NSF (*N*-ethylmaleimide-sensitive factor) attachment protein receptor
TMD: transmembrane domain
TM-N: N-terminal half of an intramembrane protease substrate TMD
TM-C: C-terminal half of an intramembrane protease substrate TMD
TFE: trifluoroethanol
TFE/water: TFE/water in buffered aqueous solution
